# Theoretical Analysis of S, M and N Structural Proteins by the Protein–RNA Recognition Code Leads to Genes/proteins that Are Relevant to the SARS-CoV-2 Life Cycle and Pathogenesis

**DOI:** 10.3389/fgene.2021.763995

**Published:** 2021-09-29

**Authors:** Jozef Nahalka

**Affiliations:** ^1^ Institute of Chemistry, Centre for Glycomics, Slovak Academy of Sciences, Bratislava, Slovakia; ^2^ Institute of Chemistry, Centre of Excellence for White-green Biotechnology, Slovak Academy of Sciences, Nitra, Slovakia

**Keywords:** computational method, COVID-19, SARS-CoV-2, identified genes, protein-RNA recognition code

## Abstract

In this conceptual review, based on the protein–RNA recognition code, some theoretical sequences were detected in the spike (S), membrane (M) and capsid (N) proteins that may post-transcriptionally regulate the host genes/proteins in immune homeostasis, pulmonary epithelial tissue homeostasis, and lipid homeostasis. According to the review of literature, the spectrum of identified genes/proteins shows that the virus promotes IL1α/β–IL1R1 signaling (type 1 immunity) and immunity defense against helminths and venoms (type 2 immunity). In the alteration of homeostasis in the pulmonary epithelial tissue, the virus blocks the function of cilia and the molecular programs that are involved in wound healing (EMT and MET). Additionally, the protein–RNA recognition method described here identifies compatible sequences in the S1A-domain for the post-transcriptional promotion of PIKFYVE, which is one of the critical factors for SARS-CoV-2 entry to the host cell, and for the post-transcriptional repression of xylulokinase XYLB. A decrease in XYLB product (Xu5P) in plasma was proposed as one of the potential metabolomics biomarkers of COVID-19. In summary, the protein–RNA recognition code leads to protein genes relevant to the SARS-CoV-2 life cycle and pathogenesis.

## Introduction

Life began with the emergence of nucleic and amino acid polymers, before the emergence of more complex proto-cells (Auboeuf, 2020), and the origins of viruses are intertwined with those of ancient proto-cells (Mughal et al., 2020). In light of this, protein–RNA recognition and protein–RNA interactions probably provide the basic rules for the communication between viruses and the host cells. In the early prebiotic stage, there was likely a stereochemical era during the evolution of the genetic code, with direct interactions between nucleotides and amino acids (Yarus et al., 2009; Yarus, 2017). It seems that glycine, alanine, aspartic acid, and valine (G, A, D, and V, respectively), which are coded with GNC codons, were the first amino acids in the first peptides/proteins (Ikehara, 2009, 2016). Taken together, and after the crystal structures of aminoacyls transfer RNA synthetases, which interact with tRNA, it was stated that the protein–RNA recognition code can be derived from the present genetic code (Nahalka, 2011, 2014; Nahalka et al., 2015).

**FIGURE 1:**
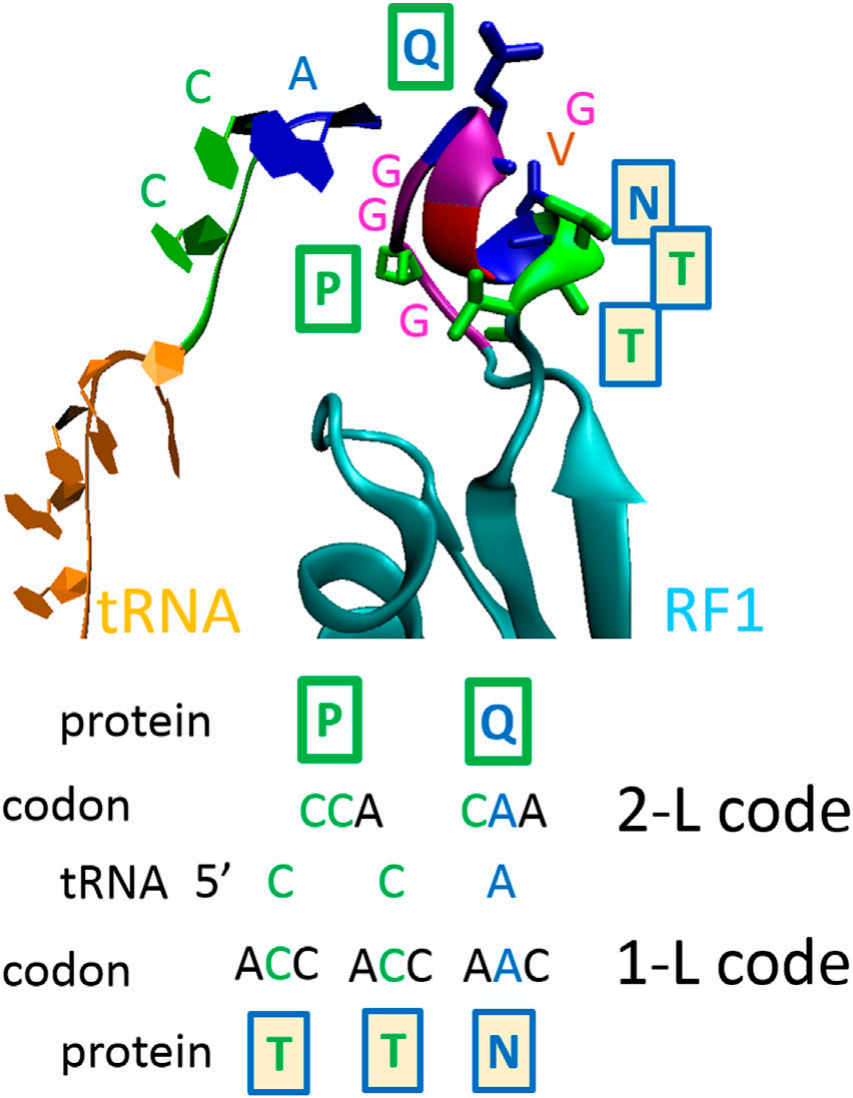
The principle of the proposed protein–RNA recognition code. One-letter code—second nucleotide in codons; two-letter code—first two nucleotides in codons (Nahalka, 2014, 2019). The ribosomal release factor 1 (RF1) interacting with P-site tRNA (3D5A). At a shorter distance, proline 227 (P) and glutamine 230 (Q) recognize 5’CCA nucleotide sequences using the 2-L code; at a longer distance, asparagine 233 (N), threonine 234 (T) and T235 recognize 3’ACC nucleotide sequences using 1-L code. In the RF1s of all organisms, the GGQ motif is inserted into the peptidyl transferase center and is positioned to contribute directly to peptidyl-tRNA hydrolysis (Laurberg et al., 2008). The GGQ motif was confirmed recently for the recognition of ribose (Nahalka and Hrabarova, 2021), which is the sugar component of RNA.

The principle of the protein–RNA recognition code is explained in Figure 1 and is depicted on the crystal structure of the ribosomal release factor 1 (RF1), which interacts with the P-site tRNA (3D5A). By recognizing a stop codon, RF1 promotes the hydrolysis of the peptidyl-transfer RNA linkage. In type I release factors of all organisms, glutamine 230 in the GGQ motif, which is the universally conserved motif, is inserted into the peptidyl transferase center and is positioned to contribute directly to peptidyl-tRNA hydrolysis and translation termination on the 70S ribosome (Laurberg et al., 2008). Interestingly, the same GGQ motif was recently proposed for the recognition of ribose (Nahalka and Hrabarova, 2021), which is the sugar component of RNA. Q230, which has CA(A/G) codons, recognizes and interacts with the C_2452_A_2451_ of rRNA and the A_76_ of tRNA (Laurberg et al., 2008). In the RF1 of *Thermus thermophilus*, in addition to Q_230_, the proline 227 (P) and NTT (asparagine-threonine-threonine) motifs help to recognize the P-site tRNA (Figure 1). In that case, the tRNA CCA_76_-end sequence readout is performed by P and Q amino acids that have CC(A) and CA(A) codons, and by T, T and N amino acids that have ACC, ACC and AAC codons (Figure 1). The CCA_76_ readout is performed by CC and CA readout-2-letter code (2-L), or by the C plus C plus A readout-1-letter code (1-L), as was proposed previously (Nahalka, 2014, 2019).

The code is currently theoretical; however, its application in the pathology of neuro-degenerative disorders has uncovered promising details (Nahalka, 2019), which indicate that the code could be used for the identification of influenced host factors/genes. For example, a miRBase search for micro-RNAs (Nahalka, 2019), obtained by the code transcription of a prion amino acid sequence, revealed new micro-RNAs and miRNAs that have previously been identified as involved in prion diseases (Nahalka, 2019). The protein–RNA recognition code was used in this study as a bioinformatics tool to search the human transcriptome. Using the protein–RNA 1-L code, three out of four SARS-CoV-2 structural proteins were transcribed to the imaginary RNA sequences and the imaginary sequences were applied to the BLASTn-search in the human transcriptome in order to reveal the compatible RNA sequences of the human host cells. According to the review of literature, the search led to genes/proteins that are relevant to the SARS-CoV-2 life cycle and pathogenesis. It is proposed in this study that viral protein–host cell RNA interactions can be implemented in the viral regulation of host genes on a post-transcriptional level.

To date, three coronaviruses have crossed the species barrier and have caused deadly pneumonia in humans: severe acute respiratory syndrome coronavirus (2002, SARS-CoV; de Wilde et al., 2018), Middle-East respiratory syndrome coronavirus (2012, MERS-CoV; Arabi et al., 2017), and severe acute respiratory syndrome coronavirus 2 (2019, SARS-CoV-2; Gordon et al., 2020). SARS-CoV-2 is known worldwide; it has caused the COVID-19 pandemic (Coronavirus disease 2019). The development of coronavirus pathology was quite unexpected, because the coronavirus family includes four “established” human coronaviruses (1960, HCoV-OC43 and HCoV-229E; 2004–2005, HCoV-NL63 and HCoV-HKU1), which cause mild respiratory disease, and after rhinoviruses, are a leading cause of common colds (de Wilde et al., 2018). The factors that trigger severe illnesses in individuals infected with SARS-CoV-2 are not completely understood, but it does not seem to be solely related to viral loads, and an excessive inflammatory response to SARS-CoV-2 is thought to be a major cause of disease severity and death in patients with COVID-19 (Merad and Martin, 2020). Naturally, the first research papers have led to the characterization of the spike glycoprotein of SARS-CoV-2 for virus entry and the inhibition of this entry (Hoffmann et al., 2020; Walls et al., 2020; Xia et al., 2020); however, a better understanding of the host factors that are influenced by SARS-CoV-2 replication is needed. For example, SARS-CoV-2 proteins were cloned in human cells, and a SARS-CoV-2 protein interaction map has been constructed (Gordon et al., 2020). However, it would be naive to think that the virus only functions at the protein–protein interactome level, as it also affects the protein-RNA and RNA-RNA interactomes.

In this paper, three out of four SARS-CoV-2 structural proteins were theoretically analyzed: QHD43416, spike protein (S); QHD43419, membrane protein (M); and QHD43423, capsid protein (N). The majority of the identified genes/proteins alter immune homeostasis, pulmonary epithelial tissue homeostasis, and lipid homeostasis. In light of biological realism in the potential of protein-RNA interactions in the cell, it has to be emphasized that upon replication of vRNA in the cytoplasm and synthesis of structural proteins in the ER, coronaviruses perform RNA-encapsidation, capsid enveloping and membrane budding into the lumen of ERGIC. However, structural viral proteins are not used quantitatively to build the virions, for example, only a small portion of E is incorporated into the viral envelope of virions (Schoeman and Fielding, 2019). They are also involved in other aspects of the replication cycle and possess innate immunity-modulating activities (de Wilde et al., 2018). In light of protein-RNA interactions in the cell, cytosolic non-structural/accessory proteins of SARS-CoV-2 would be much better and more appealing to study, however, the structural proteins were chosen here because they are currently better characterized. The accessory proteins of SARS-CoV-2 will be targeted in my next study.

## Methods

The search by the 1-L code can be performed as follows: *1*) First, transcription of proteins to the imaginary nucleotide sequence is performed, using the 1-L code, and then *2*) a blastn-search of human transcriptome is performed to identify genes.

(i) Each amino acid is transcribed to the second nucleotide of its codons, only S (Ser) has two possibilities, there is a transcription to C (cytidine) or G (guanosine), and so two nucleotide sequences are obtained for each amino acid sequence, one with S-C-transcription and the other with S-G-transcription. The second fact is that the protein–RNA readout can be performed in two directions: N-(AA)n-C versus 5’-(N)n-3’ and C-(AA)n-N versus 5’-(N)n-3’ (Figure 1), so the transcription is done for two amino acid sequences, one for N-(AA)n-C and the second for C-(AA)n-N. In summary, four nucleotide sequences are obtained (Supplementary sequence information).
(ii) For each viral protein, a blastn-search in the human transcriptome was done as a standard nucleotide blast in NCBI (https://blast.ncbi.nlm.nih.gov/Blast.cgi) for four nucleotide sequences separately. “Genomic + transcript databases” and “Human genomic plus transcript, “somewhat similar sequences” (blast algorithm), word size 7, max target sequences 500, and an expected threshold of 100 were found as optimal conditions for the search. On average, significant alignments had the size of miRNAs, and there were 18–28 nucleotides. At these conditions, 65 hits were found for the S, M and N proteins of SARS-CoV-2. The hits were divided into two groups: alignments with the reverse complement sequences (yellow in figures) and alignments with the gene transcript sequence (green in figures). Alignments with the gene transcript sequence were considered to be repressive (green in the figures), and alignments with the reverse complement sequences were considered to be promotive (yellow in the figures).

## Results

Throughout the whole study, it was considered that the contents of the viral structural proteins sequences were compatible with the mRNA of the protein host genes or with the host regulation microRNAs, and this was based on the protein–RNA recognition code. If the viral protein (or its degradation peptide) interferes with the host mRNA, then it represses translation. It is assumed that alignments with complement sequence are the sequences that are responsible for post-transcriptional repression. Micro-RNAs (MIRs) are small endogenous RNAs that pair and bind to sites on mRNAs in order to direct post-transcriptional repression (Nahalka, 2019). If the viral protein (or its degradation peptide) interferes with the host regulation MIR, then it promotes translation. It is assumed that alignments with reverse complement sequences are the sequences that are responsible for post-transcriptional promotion. Taken together, throughout the whole study, it was considered that the “primitive” primordial “protein–RNA recognition code” is currently operating in cells and is especially used in virus–host cell interactions in post-transcriptional regulation of the host cell mRNA.

### QHD43416, Spike Protein S

**FIGURE 2:**
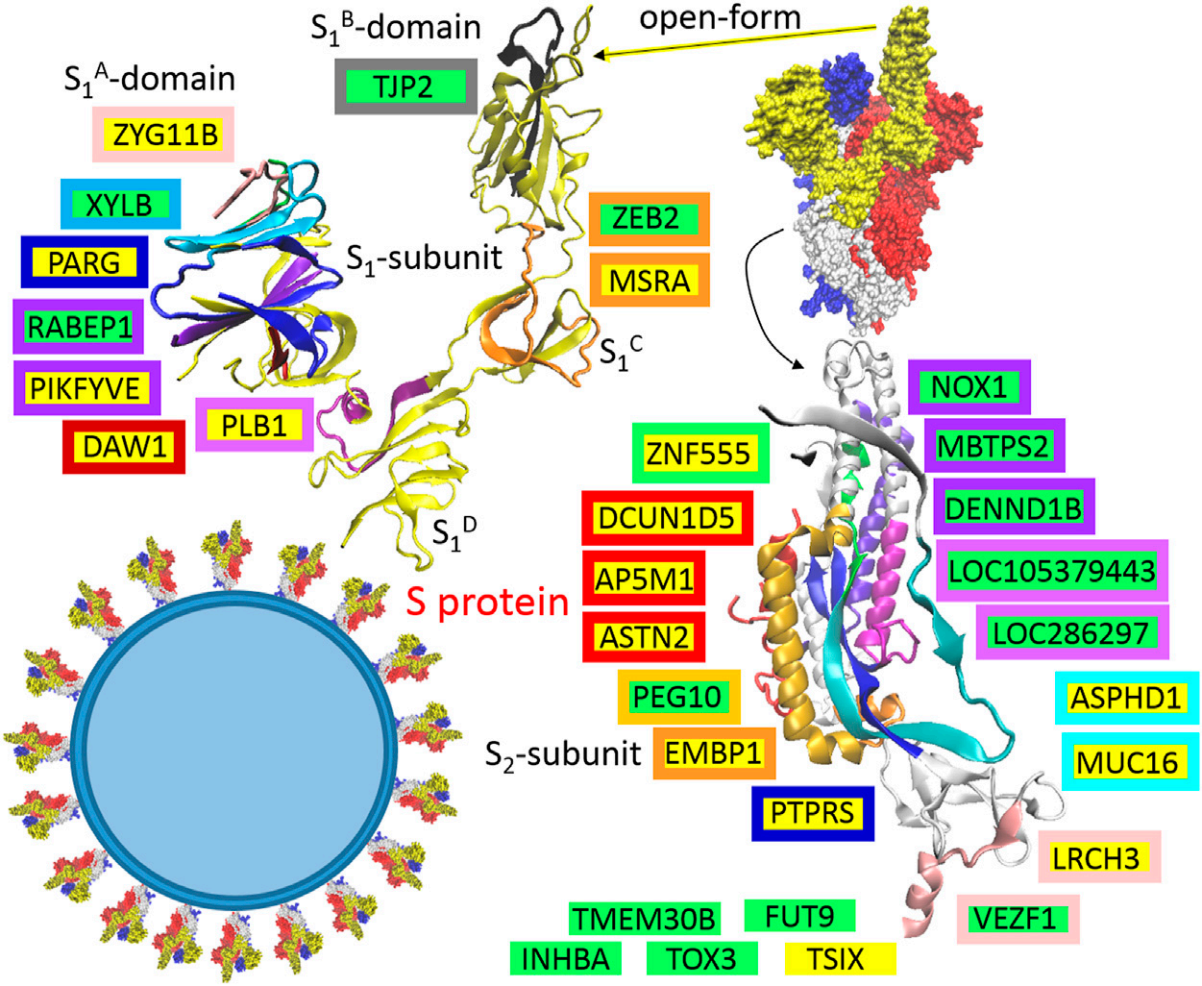
The identified sequences in QHD43416 (spike protein: S). Green highlights show the alignments with the complement sequence (post-transcriptionally repressed); yellow highlights show the alignments with the reverse complement sequence (post-transcriptionally promoted). The position in the 3D structure (6VYB; Walls et al., 2020) is colored in the same color as the frame of the gene.

S protein is the largest SARS-CoV-2 structural protein (1273 AA) that mediates binding to host cells, followed by membrane fusion. It forms homotrimers that have characteristic club-shaped forms that are projected from the envelope surface, which in electron micrographs create a circular image that is reminiscent of a solar corona, from which the virus derives its name (Figure 2). SARS-CoV-2 S glycoprotein harbors a furin cleavage site at the boundary between the S1 (residue 13–685) and S2 (residue 686–1273) subunits, which are processed during biogenesis, and the subunits remain non-covalently bound in the pre-fusion conformation (Figure 2), (Walls et al., 2020). S1 is responsible for binding to the host cell receptor (ACE2, angiotensin-converting enzyme 2), and S2 is responsible for the fusion of viral and cellular membranes (Walls et al., 2020). The distal S1 subunit comprises S1A (NTD, the N-terminal domain), S1B (RBD, the receptor-binding domain), S1C and S1D domains, which contribute to stabilization of the pre-fusion state of the membrane-anchored S2 subunit that contains the fusion machinery (Walls et al., 2020). Based on the presented theory, in the host or defense cells, S protein post-transcriptionally promotes 15 genes: PARG, DAW1, ZYG11B, MSRA, PLB1, PIKFYVE, LRCH3, ASPHD1, TSIX, DCUN1D5, AP5M1, ASTN2, MUC16, PTPRS, and ZNF555, and represses 15 other genes: ZEB2, RABEP1, TJP2, XYLB, TOX3, INHBA, VEZF1, PEG10, TMEM30B, LOC105379443, LOC286297, NOX1, FUT9, MBTPS2, and DENND1B. Localization of the responsible sequences is depicted in Figure 2. The main compatible sequences are located in the S2 subunit. In the S1 subunit, the compatible sequences are mainly concentrated in the S1A-domain. If are known, the functions of identified genes/proteins are summarized in Supplementary Table S1.

### QHD43419, Membrane Protein M

**FIGURE 3:**
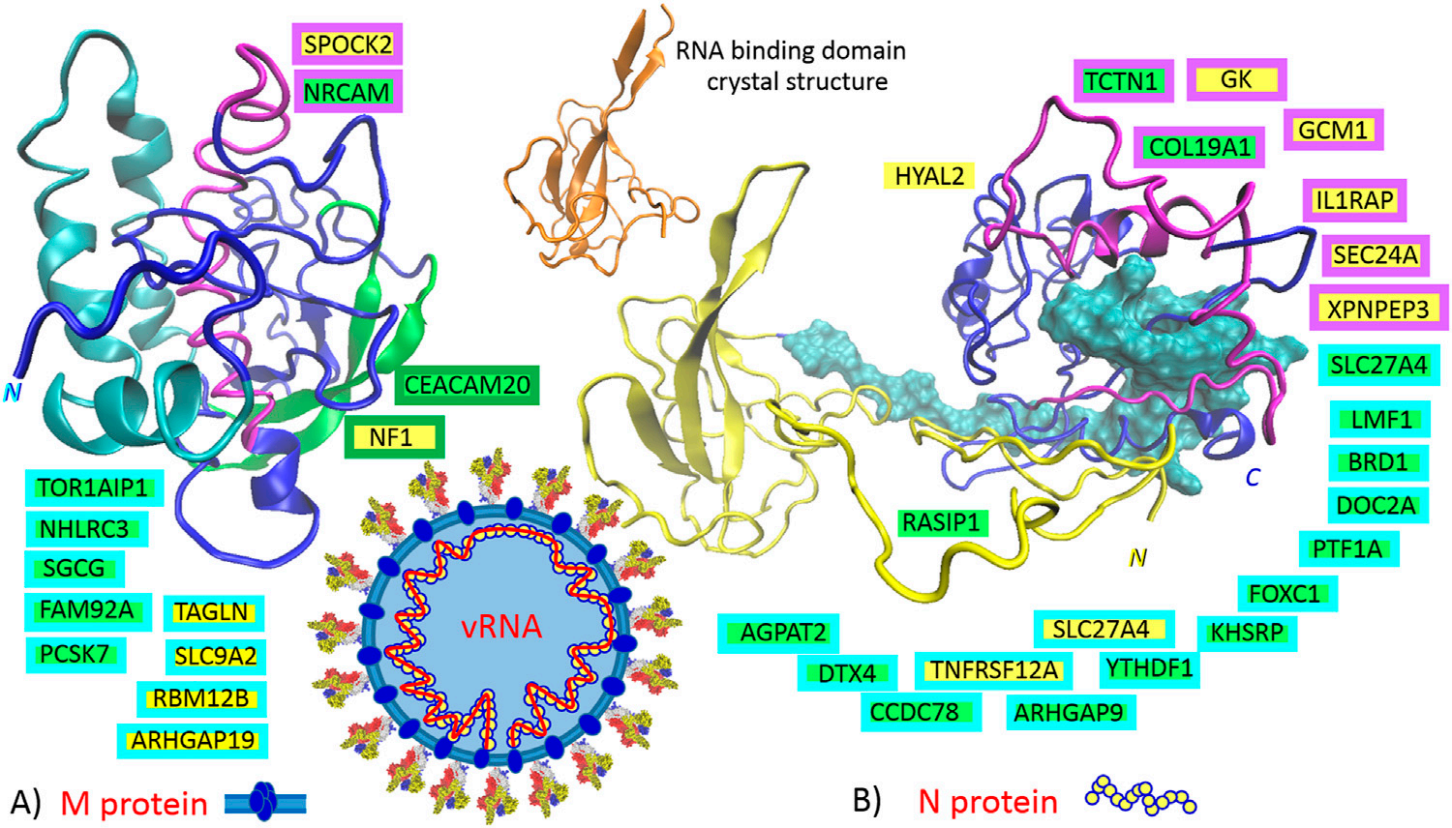
The identified sequences in QHD43419 (A., membrane protein: M) and QHD43423 (B., capsid protein: N). Green highlights show the alignments with the complement sequence (post-transcriptionally repressed); yellow highlights show the alignments with the reverse complement sequence (post-transcriptionally promoted). The position in the 3D structure models (https://zhanglab.ccmb.med.umich.edu/COVID-19/) is colored in the same color as the frame of the gene. 6M3M, N protein, crystal structure of RNA binding domain (range: 50–174), (Kang et al., 2020).

Based on the presented theory, in the host or defense cells, the M protein post-transcriptionally promotes six genes: TAGLN, SLC9A2, ARHGAP19, NF1, SPOCK2, and RBM12B, and represses seven other genes: FAM92A, PCSK7, NHLRC3, CEACAM20, SGCG, NRCAM, and TOR1AIP1. Localization of the responsible sequences is depicted in Figure 3A. The most compatible sequences are located in the N-terminal “finger”-structured region of the protein (Figure 3A, M protein, “finger”-structured region in cyan). If are known, the functions of identified genes/proteins are summarized in Supplementary Table S1.

### QHD43423, Capsid Protein N

The domain architecture of the coronavirus N protein consists of three distinct but highly conserved parts: an N-terminal RNA-binding domain (NTD, yellow in Figure 3B), a C-terminal dimerization domain (CTD, blue in Figure 3B), and an intrinsically disordered central S/R-rich linker (cyan in Figure 3B); the NTD is responsible for RNA binding, the CTD is for oligomerization, and the (SR)-rich linker is for primary phosphorylation (Kang et al., 2020). Based on the presented theory, in the host or defense cells, N protein post-transcriptionally promotes eight genes: GK, GCM1, SLC27A4, HYAL2, TNFRSF12A, IL1RAP, SEC24A, and XPNPEP3, and represses 14 other genes: TCTN1, LMF1, BRD1, DOC2A, PTF1A, FOXC1, KHSRP, ARHGAP9, YTHDF1, CCDC78, RASIP1, DTX4, AGPAT2, and COL19A1. An intrinsically disordered S/R-rich linker (Figure 3B, N protein, disordered central linker in cyan) locates the main portion of compatible sequences, which repress the genes. The sequences that promote the genes are mainly localized at the C-end of CTD (Figure 3B, N protein, CTD in purple). Interestingly, no compatible sequence was found in the N-terminal RNA-binding region (NTD, residues 46 to 174, “finger” in yellow), and there is only one compatible sequence in the disordered region of the N-terminus (residues 14–40, RASIP1). If are known, the functions of identified genes/proteins are summarized in Supplementary Table S1.

## Discussion

The SARS-CoV-2 protein interactome revealed aspects of SARS-CoV-2 biology and potential pharmacological targets (Gordon et al., 2020); however, the virus is a very small “molecular program,” and it is essential for it to use every possible type of molecular interaction in order to hijack the host cell molecular machinery; thus, the virus must also be programmed at the level of the protein–RNA and RNA-RNA interactomes. In this study, the theoretical protein–RNA recognition code was used for bioinformatics identification of the interactions between structural proteins of SARS-CoV-2 and the host cell transcriptome. At present, it is difficult to determine whether the viral proteins or the degradation peptides from the viral proteins recognize and interact with the host-cell RNA in order to post-transcriptionally modify the translation of host-cell proteins; however, the identified genes are clearly involved in the SARS-CoV-2 life cycle and pathogenesis. In the next section, the identified genes are comprehensively reviewed and discussed.

### SARS-CoV-2 Life Cycle in the Host Cells

**FIGURE 4:**
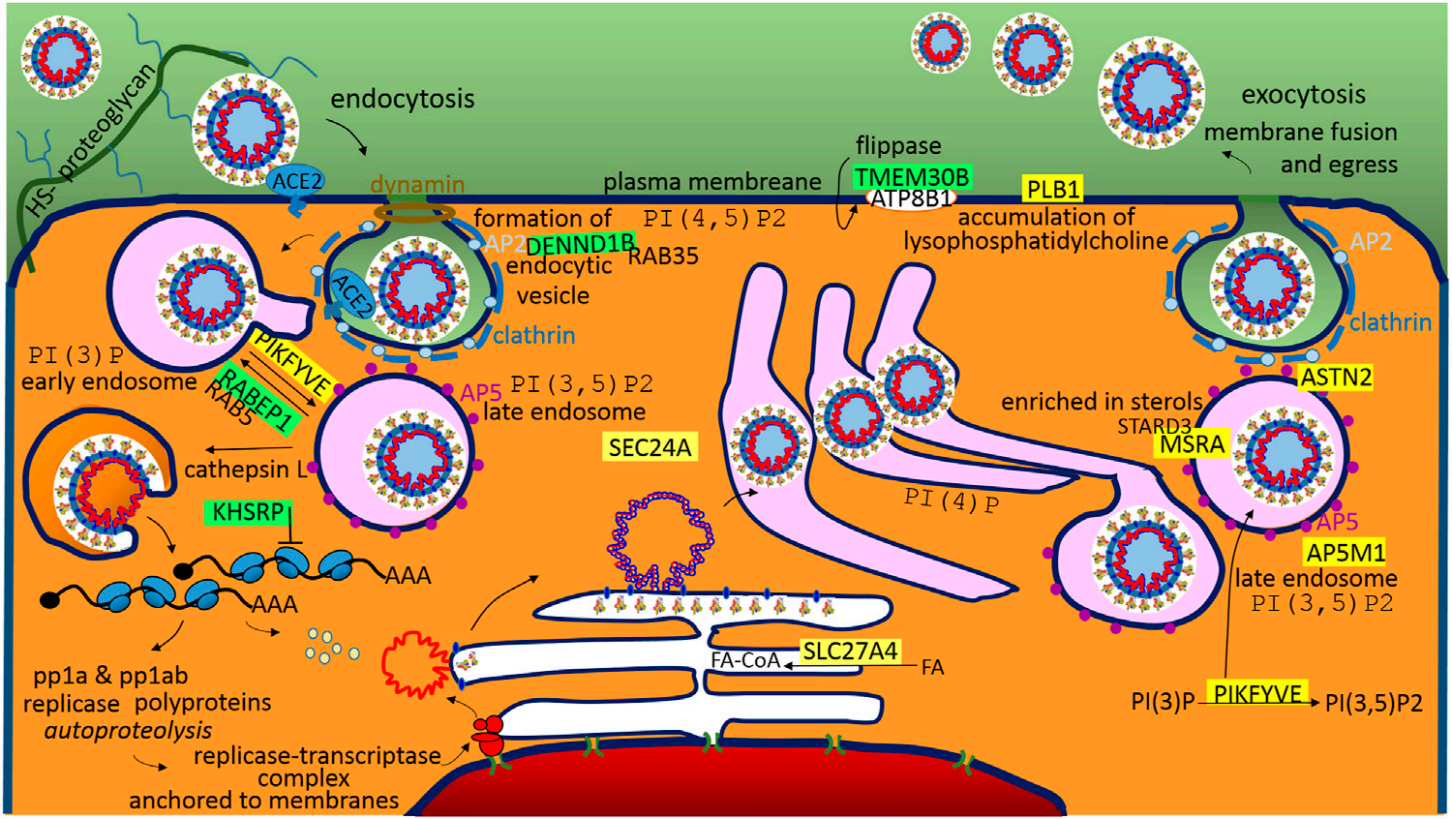
A graphical illustration of “SARS-CoV-2 life cycle in the host cells.” Green highlights show the alignments with the complement sequence (post-transcriptionally repressed); yellow highlights show the alignments with the reverse complement sequence (post-transcriptionally promoted). The functions of the identified genes/proteins by the methodology are summarized in Supplementary Table S1.

It is generally accepted that SARS-CoV-2 cell entry depends on the accessibility of ACE2 and TMPRSS2 (Hoffmann et al., 2020); ACE2 is an interferon-stimulated angiotensin-converting enzyme 2 used as a binding receptor for the S protein (Ziegler et al., 2020), and TMPRSS2 is the transmembrane protease-serine subfamily member 2 used for the S2 subunit-priming for membrane fusion (Tang et al., 2020) (Figure 4). In coronaviruses, a general rule of thumb is that the S1A-domain of the S1-subunit is associated with viral binding to a sugar-based receptor (heparan sulfate-proteoglycan) and that the S1B-domain is associated with binding to a protein receptor, ACE2, in the cases of SARS-CoV and SARS-CoV-2 (Figure 4). In both SARS viruses, S-protein binding to ACE2 requires one S monomer in the trimers to be in the “up” position, and after receptor binding, both viruses can use the plasma membrane fusion pathway (membrane protease, such as TMPRSS2) or endosomal fusion pathway (endolysosomal acidification and proteases) in order to allow for S2 subunit-priming and to trigger viral plasmatic/endosomal membrane fusion (Tang et al., 2020). However, SARS-CoV-2 mainly enters 293/hACE2 cells through endocytosis (Ou et al., 2020), and the virus traffics through the endolysosomal system. It seems that PIKFYVE is other factor critical for SARS-CoV-2 entry (Ou et al., 2020). Interestingly, the protein–RNA recognition method described here identified a compatible sequence in the S1A-domain for the post-transcriptional promotion of PIKFYVE and post-transcriptional repression of RABEP1 (Figure 2). PIKFYVE (phosphoinositide kinase FYVE-type) synthesizes phosphatidyl-inositol-3,5-bisphosphate (PI(3,5)P2; Ou et al., 2020) and RABEP1 (RAB GTPase binding effector protein 1) functions as a vital activator and molecular switch for RAB5, an early endosome associated GTPase (Ding and Xiang, 2018), which positively regulates PI(3)P synthesis (Di Paolo and De Camilli, 2006). PI(3)P is a major determinant of early endosome membrane identity, while PI(3,5)P2 is a major determinant of late endosome membrane identity (Di Paolo and De Camilli, 2006), (Figure 4). Of the cargo internalized by ongoing endocytosis in mammalian cells, the majority are recycled back to the plasma membrane *via* early endosomes (Huotari and Helenius, 2011), so it seems important for the virus entry to shift early endosome maturation to the late endosomes; in light of this, the promotion of PIKFIVE and the blocking of RABEP1-RAB5 complex seem to make sense.

The S2-subunit provides a sequence for the post-transcriptional promotion of AP5M1, which is the µ subunit in the adaptor protein complex 5 (AP5). Adaptor protein (AP) complexes are heterotetramers that shape membranes into vesicles and select cargo for inclusion into transport vesicles. The function of AP5 is to retrieve a number of proteins from late endosomes and to recycle them to the Golgi complex (Sanger et al., 2019). The S2-subunit also provides a sequence for the post-transcriptional repression of DENND1B, which is a guanine nucleotide exchange factor (GEF) that interacts with RAB35 and AP2 (Yang et al., 2016). RAB35 is a regulator of the phosphatidylinositol 3′-OH kinase (PI3K) pathway (Wheeler et al., 2015), and AP2 (AP-complex 2) is an abundant component of clathrin-coated vesicles that is located at the plasma membrane (Sanger et al., 2019), (Figure 4). Both AP2 and AP5 appear to be distinct from other AP-complexes, and they do not use small GTPase ARF1 for the recruitment into the membrane, but have a strong phosphoinositide requirement, making use of PI(4,5)P2 (AP2-plasma membrane), PI(3)P (AP5-early endosome), or the abovementioned PI(3,5)P2 produced by PIKFYVE (AP5-late endosome). AP5 is closely associated with spastizin (SPG15) and spatacsin (SPG11) forming the AP5–SPG11–SPG15 complex (Huotari and Helenius, 2011). Interestingly, SPG11 controls cholesterol trafficking in the endolysosomal pathway, and the loss of spatacsin function and the associated inhibition of tubule formation lead to a decrease in cholesterol in the plasma membrane and, consequently, to the accumulation of cholesterol in the late endosome–lysosome (Boutry et al., 2019). StAR-related lipid transfer domain containing three (STARD3) mediates the transport of cholesterol from the endoplasmic reticulum to the endosome (Lim et al., 2018). Interestingly, the S1C domain has a sequence that supports the post-transcriptional promotion of MSRA, which is a binding partner of STARD3. MSRA (methionine sulfoxide reductase A) protects endosomal proteins from oxidative damage, and as was indicated, may play a role in lipid metabolism. ASTN2 (astrotactin 2) is the next promoted endo-lysosomal protein, which is involved in trafficking and the degradation of surface proteins (Behesti, et al., 2018). Interestingly, it binds the clathrin adaptor AP2 (Behesti, et al., 2018).

Following entry of the virus, S2 subunit-priming, membrane fusion and releasing of the genome, the coronavirus replicative cycle starts with the translation of the 5′-proximal ORFs (ORF1a and ORF1b), which results in the synthesis of two large replicase polyproteins (pp1a and pp1ab). After proteolytic processing, together with recruited host cell proteins, the coronavirus forms membrane-associated replication and transcription complexes in the perinuclear region (de Wilde et al., 2018), (Figure 4). The machinery employs a unique transcription mechanism in order to generate a nested set of subgenomic mRNAs (de Wilde et al., 2018). The N protein provides a sequence that post-transcriptionally represses KHSRP (Figure 4). KHSRP, a KH-type splicing regulatory protein, belongs to the proteins that recognize the AU-rich element (ARE) in the 3′-untranslated regions (UTRs) of mRNAs to control factors that require very tight regulation; in the cytoplasm, KHSRP negatively regulates cap-independent translation processes (Otsuka et al., 2019). Internal ribosome entry sites (IRESs) are functional RNA elements that can directly recruit ribosomes to an internal position of the mRNA in a 5’ cap-independent manner in order to initiate translation (Zhao et al., 2020). Taken together, IRES-driven translation of SARS-CoV-2 mRNAs may be essential. In SARS-CoV, the nsp1 protein inhibits translation of host IRES-driven mRNAs, while SARS coronavirus mRNAs are resistant to nsp1-induced RNA cleavage (Lokugamage et al., 2012).

Upon replication and synthesis of structural proteins in the ER, it is accepted that CoV RNA-encapsidation, membrane budding and capsid enveloping are performed in ERGIC, which is the ER-Golgi intermediate compartment (de Wilde et al., 2018). The N protein provides sequences that post-transcriptionally activate SLC27A4 and SEC24A. SLC27A4/FATP4, which is fatty acid (FA) transport protein 4, is an acyl-CoA synthetase that provides energy for the structural protein synthesis in ER, activates FAs and channels them towards oxidation (Grevengoed et al., 2014). The SEC24 homologue A is the coat protein complex II (COPII) component, which mediates transport from the ER to the Golgi; the inner layer SAR1 and SEC23–SEC24 heterodimer bind to and select specific cargo for packaging into ER-derived transport vesicles, which indicates that A from four available isoforms of SEC24A-D is the interaction partner of SARS-CoV-2.

After maturation in the Golgi, the virus follows endosomal-exocytosis (Figure 4). The S1A-domain supports the promotion of PLB1 (phospholipase B1), which stimulates the initiation of sperm acrosome exocytosis and membrane fusion (Asano et al., 2013). PLB1 is activated in response to a sterol decrease (Asano et al., 2013). Interestingly, the S2 subunit has a sequence for post-transcriptional repression of TMEM30B/CDC50B, the β-subunit of the phospholipid flippase type IV P-type ATPase family, which associates with the α-subunit ATP8B1 (Figure 4). Mutations in the ATP8B1 are responsible for liver cholestasis (BRIC1, PFIC1), and affected individuals are more susceptible to pneumonia (Andersen et al., 2016).

### Altered Immune Homeostasis as a Consequence of SARS-CoV-2 Infection

**FIGURE 5:**
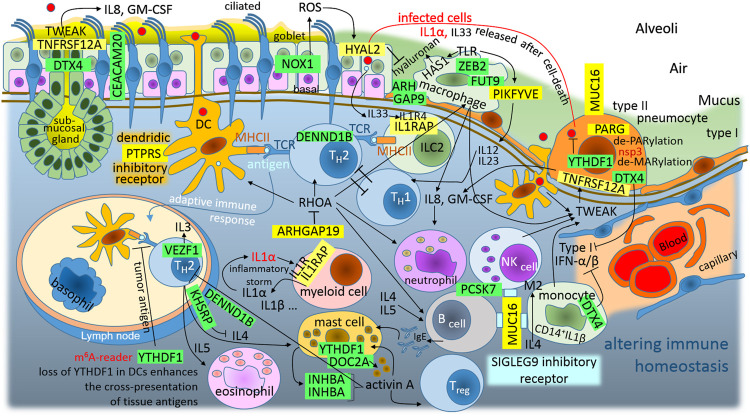
The graphic illustration of “Altered immune homeostasis is a consequence of SARS-CoV-2 infection.” Green highlights show the alignments with the complement sequence (post-transcriptionally repressed); yellow highlights show the alignments with the reverse complement sequence (post-transcriptionally promoted). The functions of the identified genes/proteins by the methodology are summarized in Supplementary Table S1.

The morbidity and mortality levels observed in COVID-19 are associated with excessive inflammation, and a better understanding of the differential responses seen in patients infected with SARS-CoV-2 is necessary in order to identify therapeutic targets (Merad and Martin, 2020) (Figure 5). The human immune responses against pathogens and in allergy/hypersensitivity include the various types of immune pathways, have quite wide spectrum of the immune cells, and are variable for each person; however despite the problematic categorization, it is useful organize immune pathways logically into a summary diagram. In the traditional view, we have three basic lines: type 1 immunity against intracellular pathogens (TH1-defence, viruses and intracellular bacteria, production of IFNγ-cytokine, inflammation/autoimmunity); type 2 immunity against multicellular parasites (TH2-defence, production of IL4 and IL5 cytokines, allergy/asthma); and type 3 immunity against extracellular pathogens (TH17-defence, bacteria and fungi, production of IL22 and IL17 cytokines, inflammation/autoimmunity), (Annunziato et al., 2015). Recently this model was reclassified to the 4 × 2 + 2 immunological pathways (Hu, 2020); however, to simplify the things, the results are discussed here in the reference to the above mentioned basic three line model. Theoretical post-transcriptional dysregulation of immune genes in bronchoalveolar tissue, identified by this simple method, is depicted in Figure 5.

Among airway tissue-resident cell subsets, lung alveolar type II pneumocytes and bronchial goblet cells are the main targets of SARS-CoV-2 infection (Ziegler et al., 2020). These infected cells release IL1α after their death, alarmin IL1α signals *via* IL1R on myeloid cells induce the expression of pro-inflammatory cytokines; this signaling further leads to the expression of cytokines and chemokines, including IL1α and IL1β, which leads to the recruitment of inflammatory cells to the site of the damage (Malik and Kanneganti, 2018). Therefore, the initial round of IL1α–IL1R1 signaling can lead to self-perpetuating inflammation–inflammatory storm (Malik and Kanneganti, 2018). IL1α–IL1R1 signaling needs the IL1RAP coreceptor, and SARS-CoV-2 N-protein promotes this coreceptor (Figure 3B). The N-protein represses YTHDF1/DF1, which is the N6-methyladenosine (m6A)-modification RNA reader, and its loss in classical dendritic cells enhances the cross-presentation of tissue antigens and elevates antigen-specific CD8^+^ T cell antitumor responses (Han et al., 2019). It activates T helper 2 (TH2) cells, which orchestrate protective type 2 immune responses, such as those that target helminths and facilitate tissue repair, but also contribute to chronic inflammatory diseases (Walker and McKenzie, 2018). In addition, IRES trans-acting factor KHSRP, which is repressed with the N protein, regulates the stability of many mRNAs encoding immune-relevant proteins, and has been described as a negative regulator of IL4 expression; T cells derived from KSRP−/− mice favor TH2-driven immune responses (Käfer et al., 2019). Additionally, the S2-subunit post-transcriptionally represses DENND1B, which is an important GEF for the down-modulation of the T cell receptor in TH2 cells, and Dennd1b−/− mice exhibit >3-fold increases in the production of IL4, IL5, and IL13 (Yang et al., 2016). This contributes to monocyte-M0-macrophage polarization to an M2-like phenotype (IL4), mast cell activation (IL4), B cell activation (IL4 and IL5), and eosinophil activation (IL4). Lung mast cells are an important source of activin in the airways, and activin-A induces regulatory T cells that suppress T helper cell immune responses and protect from allergic airway disease (Semitekolou et al., 2009). Interestingly, the S2-subunit post-transcriptionally switches off this kind of feedback (closed-loop) control, and it represses INHBA, which forms activin A homodimer (Figure 5). Taken together, the repression of YTHDF1, DENND1B, KHSRP, and INHBA represents the second circle that can lead to self-perpetuating inflammation–inflammatory storm, which accelerates TH2-driven immune responses against the host-tissue (Figure 5). In addition to this inflammatory circle, infected goblet cells and type II pneumocytes also release larger quantities of IL33. IL33 is the other member of the IL1 family, which works together with IL1α as an alarmin or the molecule of damage-associated molecular signal, indicating the breach of the barrier in bronchoalveolar tissue (Boraschi et al., 2018). The group 2 innate lymphoid cells (ILC2), located at the epithelial barrier, accept the signal and present antigens to T cells in order to prime TH2-driven immune responses (Figure 5). IL33 activates IL1R4, which undergoes a conformational change that allows for the recruitment of the accessory chain IL1RAP, which is promoted by the N-protein (Figure 3B).

The M protein contains a sequence to support the promotion of ARHGAP19, Rho GTPase activating protein 19, which is predominantly expressed in hematopoietic cells and acts as a GAP for RHOA, which is the key switch of innate and adaptive immunity (Bros et al., 2019). In innate immunity, RHOA is involved in: dendritic cell and macrophage cell migration following chemoattractants, M0 macrophage polarization to M1 or M2, M-phagocytosis, and dendritic cell differentiation, DC-activation and DC-interaction with T cells. In adaptive immunity, RHOA is involved in T-cell and B-cell differentiation, migration, and activation (Bros et al., 2019). It makes sense that SARS-CoV-2 attempts to inhibit the surface macrophages and the dendritic cells, which are capable of phagocytosis and constantly sample the surrounding environment for pathogens such as viruses (Figure 5).

In the case of dendritic cells, the S2-subunit post-transcriptionally promotes PTPRS (protein tyrosine phosphatase receptor type S), which is a specific inhibitory receptor in human plasmacytoid dendritic cells (pDCs), (Bunin, et al., 2015). pDCs are a unique sentinel cell type that play important roles in antiviral immune responses (Reizis, 2019). The ligation of PTPRS inhibits pDC activation, and the ligands likely involve diverse heparan sulfate proteoglycans, suggesting a potential way for pDCs to monitor the integrity of the extracellular matrix (Reizis, 2019).

In the case of the alveolar macrophages, the S1C-domain post-transcriptionally represses ZEB2 (zinc finger E-box binding homeobox 2), which is a critical molecule for maintaining the tissue identities of macrophages. Loss of ZEB2 alters the transcriptome of macrophages, which results in changes in different macrophage populations and their subsequent disappearance (Scott et al., 2018). The S2-subunit post-transcriptionally represses FUT9 (fucosyltransferase 9), which preferentially fucosylates the distal GlcNAc residue of the polylactosamine chain (Nishihara et al., 1999). Macrophage/leukocyte adhesion to the epithelial/endothelial surface is initiated by the binding of sialolactosamine-fucosylated carbohydrates expressed on macrophages/leukocytes to epithelial/endothelial E/P-selectin, and FUT9 plays a significant role during human macrophage/leukocyte-epithelial/endothelial interactions (Buffone et al., 2013). The N protein represses ARHGAP9, which intracellularly regulates the adhesion of hematopoietic cells to the extracellular matrix (Furukawa et al., 2001). In the addition to adhesion of the alveolar macrophages to the epithelial/endothelial surface, it seems that PIKFYVE plays a role in preventing the infiltration of eosinophils and lymphoid cells through regulating alveolar macrophage function (Kawasaki et al., 2017). On the other hand, PIKFYVE (promoted by the S1A-domain), which supports lysosome fission and enables the exit from lysosomes (Saffi and Botelho, 2019), works as a critical player in toll-like receptor (TLR) signaling; for example, it is necessary for IL12 and IL23 expression (Cai et al., 2013). IL12 activates type 1 immunity (TH1-defence against viruses and intracellular bacteria, inflammation/autoimmunity, IFN-γ) and IL23 activates type 3 immunity (TH17-defence against extracellular bacteria and fungi, inflammation/autoimmunity, IL22), (Annunziato et al., 2015). IL12 has anti-angiogenic activity, which means that it can block the formation of new blood vessels (by the upregulation of CXCL10).

It seems that TH1-defence against viruses may be repressed by accelerated TH2-defence (Figure 5), while excessive tissue inflammation is supported by IL1α–IL1R1–IL1RAP inflammatory storm. However, IL12 reduces the IL4-mediated suppression of IFN-γ (type II IFN), and additionally N protein “switches off” DTX4, which is the negative regulator of type I IFN signaling and antiviral immunity. DTX4 (deltex E3 ubiquitin ligase 4) polyubiquitinates TBK1 and mediates its degradation (Cui et al., 2012). TBK1 kinase plays an important role in antiviral responses and activates the transcription factor IRF3, which leads to the induction of the type I interferon signaling that is required for viral clearance (Cui et al., 2012). Clearly, the virus supports antiviral immunity and inflammation, and the upregulation of both IFN-types.

Activated monocytes, macrophages, DCs, and NK cells secrete TWEAK/TNFSF12 (TNF-related weak inducer of apoptosis); in infected bronchoalveolar cells, the N protein promotes receptor TNFRSF12A/FN14. While TWEAK-FN14 activation is beneficial in the context of acute tissue injury, persistent activation can lead to chronic inflammation, tissue damage, and fibrosis; TWEAK-FN14 signaling is also dysregulated in autoimmune diseases (Dostert et al., 2019) and in asthma (Tessier et al., 2017). Expression of TNFRSF12A/FN14 increases after SARS-CoV infection of a human hepatoma cell line, Huh7 (Tang et al., 2005). Interestingly, a TWEAK-FN14 interaction stimulates human bronchial epithelial cells to produce IL8 and GM-CSF (granulocyte-macrophage colony-stimulating factor), (Xu et al., 2004). IL8 is known as a neutrophil chemotactic factor and is believed to play a role in the pathogenesis of bronchiolitis, a common respiratory tract disease caused by viral infection (Xu et al., 2004). Interestingly, its upregulation was also observed in IBD (inflammatory bowel disease), while CEACAM20 was downregulated (Kelleher et al., 2019). The M protein provides a sequence for the post-transcriptional downregulation of CEACAM20.

The S2-subunit post-transcriptionally represses NOX1 (NADPH oxidase 1), which is a ROS-producing oxidase. During inflammation of the colon, a strong expression of NOX1 can be observed in colon epithelial cells, NOX1 seems to be required for wound healing, and repression of the NOX1 can lead to IBD (Breitenbach et al., 2018). During inflammation of the lung caused by the influenza A virus, NOX1 suppresses inflammation and oxidative stress, and on the contrary, the NOX2 isoform seems to have a pro-inflammatory role in the regulation of influenza A infections (Selemidis et al., 2013). In IBD, it was recently demonstrated that a lowered ROS-production in cells due to the loss of NOX1 is associated with delayed wound healing, cytoskeletal changes, and altered collective cell migration, which affect tissue repair and barrier function (Khoshnevisan et al., 2020). Human bronchial epithelial (NHBE) cells express HYAL2 (hyaluronidase 2), a hyaluronan-degrading enzyme, and ROS increases HYAL2 activity and expression. In inflammatory conditions associated with oxidative stress, HYAL2 is responsible for the continuous hyaluronan fragmentation (Monzon et al., 2010). It seems that, to compensate for lower NOX1 stimuli and the lower HYAL2 activity, the N protein contains a sequence for the post-transcriptional promotion of HYAL2 (Figure 3B).

In infected cells, the S2-subunit post-transcriptionally promotes MUC16, a large transmembrane mucin that seems to have evolved to monitor and repair damaged epithelia, but these functions can be hijacked by pathogenic microbiota, viruses or cancer cells (Van Putten and Strijbis, 2017). Surprisingly, SIGLEG9 was found to be the receptor for MUC16 on human NK cells, B cells, and monocytes. SIGLEG9 is an inhibitory receptor that attenuates T cell and NK cell function (Belisle et al., 2010), for example, blocking SIGLEG9 reverses NK cell suppression (Zhao et al., 2018).

SARS-CoV-2 S glycoprotein is entirely processed at the S1/S2 site during biosynthesis in the host cells, presumably by furin in the Golgi compartment, in contrast to S glycoproteins of SARS-CoV and other coronaviruses, which incorporate into pseudovirions that are largely uncleaved and will undergo S1/S2-processing upon encountering a target cell (Walls et al., 2020). The furin-like proprotein convertases (PACE4, PC5, and PCSK7/PC7) may selectively compensate for the activation of viral glycoproteins in furin-deficient cells and tissues with different efficiencies (Garten, 2018); however, the SARS-CoV-2 M protein provides a sequence to post-transcriptionally repress PCSK7/PC7 (Figure 3A). The probable reason for this lies in the fact that high levels of PCSK7/PC7 mRNA are generally found in cells of the immune system, which indicates that the virus needs to repress the role of PCSK7/PC7, which is associated with immune responses (Garten, 2018).

In mast cells, which are immune cells of hematopoietic lineage and play an important role in inflammation, secretory granules contain lysosomal components and are therefore referred to as secretory lysosomes (Higashio et al., 2008). DOC2α (double C2 domain alpha), regulates Ca^2+^-dependent secretory lysosome exocytosis, and this exocytosis is severely reduced in bone marrow-derived mast cells from DOC2α knockout mice (Higashio et al., 2008). It seems that the N protein inhibits lung mast cell degranulation through the repression of DOC2A (Figure 3B).

In inflammation, and especially in antiviral responses, mono and poly(ADP-ribose) polymerases perform post-translational mono-ADP-ribosylation (MARylation) and poly-ADP-ribosylation (PARylation), which are involved in gene regulation and PAR-dependent ubiquitylation-protein degradation (Gupte et al., 2017). The S1A-domain post-transcriptionally promotes PARG poly(ADP-ribose) glycohydrolase, which reverses post-translational PARylation (de-PARylation). PARG degrades polymers by hydrolysis of the ribose–ribose ether bond, but cannot reverse mono-ADP-ribosylation; however, all members of *Coronavirinae* contain a highly conserved macrodomain, within nonstructural protein 3, with de-MARylation activity. The macrodomain is required to prevent PAR-mediated inhibition of coronavirus replication and the enhancement of interferon production (Grunewald et al., 2019).

### Altered Homeostasis in the Pulmonary Epithelial Tissue During the SARS-CoV-2 Infection

**FIGURE 6:**
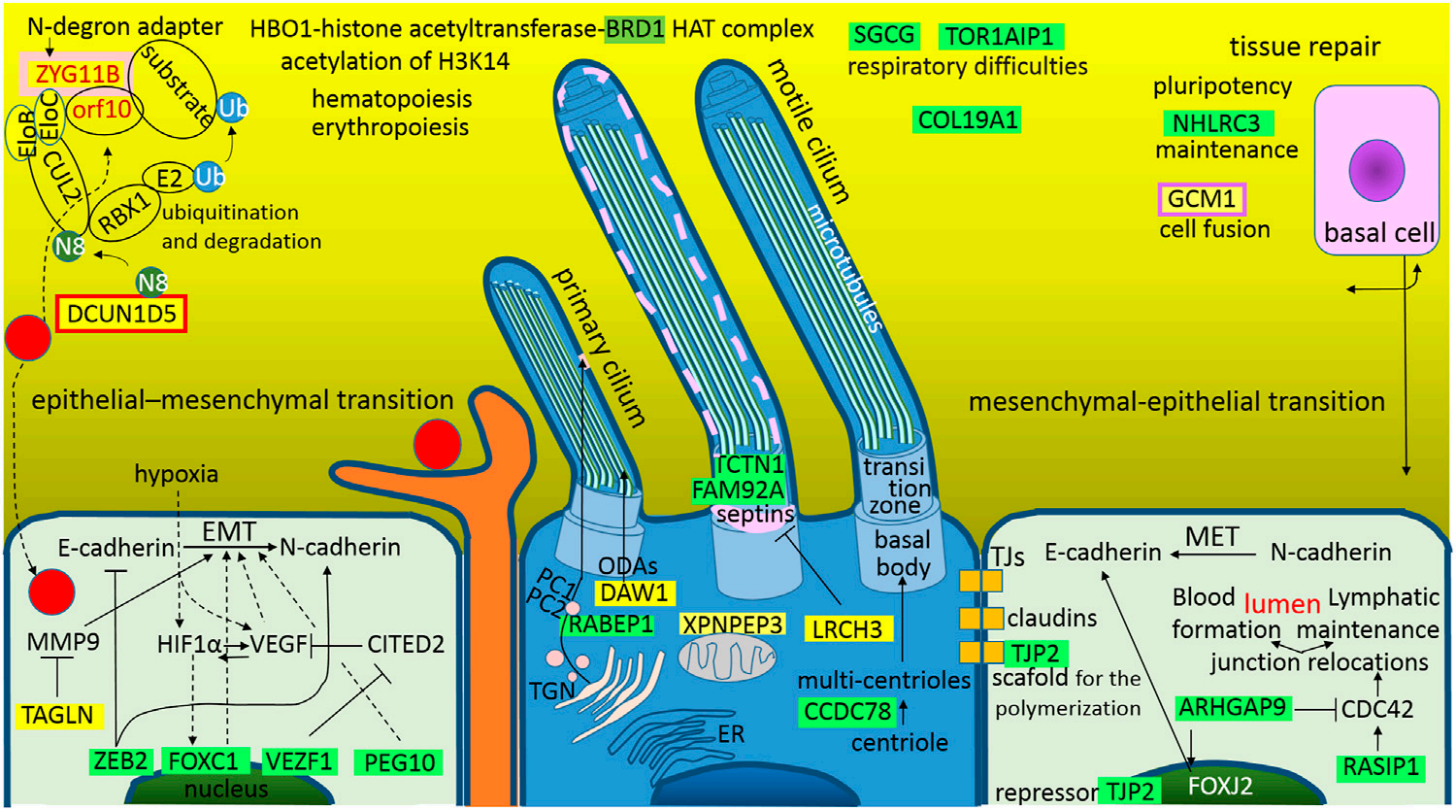
The graphical illustration of “Altered homeostasis in the pulmonary epithelial tissue during the SARS-CoV-2 infection.” Green highlights show the alignments with the complement sequence (post-transcriptionally repressed); yellow highlights show the alignments with the reverse complement sequence (post-transcriptionally promoted). The functions of the identified genes/proteins by the methodology are summarized in Supplementary Table S1.

As was depicted in Figure 5, from the trachea to the alveoli, there is a continual line of basement membranes; the human airway epithelium is composed of four major cell types: “ciliated cells, goblet cells, secretory cells, and basal cells” (Tilley et al., 2015) (Figure 6). Ciliated cells are the most characteristic feature of this epithelium; they wear motile cilia and this movement mediates mucociliary clearance of the airways from inhaled particles and pathogens (Tilley et al., 2015). In numerous lung diseases, this primary defense mechanism is disturbed by abnormalities in both the cilia movement and structure. SARS-CoV-2 is not an exception; it also affects the cilia structure and function (Figure 6). As mentioned above, the S1A-domain post-transcriptionally represses RABEP1 (Figure 2); RABEP1 is required for ciliary localization of the polycystin complex (PC1-PC2 complex controls proper tubular diameter), and the inhibition of RABEP1 causes defective ciliary function (Kim et al., 2014). The N protein post-transcriptionally represses CCDC78 (coiled-coil domain containing 78), which is a deuterosome protein that is essential for centriole amplification in multiciliated cells of the respiratory tract. This means that the inhibition of CCDC78 causes low and defective ciliary formation (KlosDehring et al., 2013; Spassky and Meunier, 2017). The N protein also has a sequence for the post-transcriptional repression of TCTN1 (tectonic family member 1), which is a protein of a transition zone complex that regulates mammalian ciliogenesis and ciliary membrane composition, and a loss of TCTN1 causes tissue-specific defects in ciliogenesis (Garcia-Gonzalo et al., 2011). TCTN1 was identified as a JBTS13 gene (Garcia-Gonzalo et al., 2011). Joubert syndrome is characterized by cerebellar and brainstem malformations, and consequently, its most common features include ataxia (lack of muscle control) and hyperpnea (abnormal breathing patterns). The M protein post-transcriptionally represses an additional protein of a transition zone complex, FAM92A (family with sequence similarity 92 member A). FAM92A colocalizes with CBY1 at the base of cilia in airway multiciliated cells; similarly to CBY1, FAM92A plays a crucial role in the formation and function of cilia, and its knockdown abrogates the formation of primary cilia (Li et al., 2016).

The S1A-domain post-transcriptionally promotes DAW1, and the S2-subunit promotes LRCH3. The upregulation of DAW1 and LRCH3 will negatively influences cilia formation and function. DAW1/ODA16 (dynein assembly factor with WD repeats) serves as an adaptor for the transport of outer dynein arms (Wang et al., 2020). LRCH3 (leucine rich repeats and calponin homology domain containing 3) forms DOCK7-LRCH3-MYO6 complex, which is the regulatory complex of septins (O'Loughlin et al., 2018). Septins associate with cellular membranes and the cytoskeleton, and play roles in numerous processes, including cytokinesis, cell polarity, and cell migration, but especially in the biogenesis and function of cilia and flagella (Palander et al., 2017). The N protein post-transcriptionally promotes XPNPEP3 (X-prolyl aminopeptidase 3), mutations in XPNPEP3 have somehow been implicated in the development of “nephronophthisis-like ciliopathy,” a cystic kidney disease (O'Toole et al., 2010; Singh et al., 2017). It seems that upregulation of this mitochondrial enzyme could also negatively influence cilia formation and function.

Epithelial cells line organs, which are exposed to the outside world, including the skin, respiratory, gastrointestinal and urogenital tracts; during development or wound healing, these cells use the epithelial-mesenchymal transition (EMT) and reverse mesenchymal-epithelial transition (MET) cellular programs, which have also been suggested to play crucial roles in the metastatic dissemination of carcinomas (Banyard and Bielenberg, 2015). One basic characteristic of these two programs is that, during EMT, the cells lose E-Cadherin, which is responsible for tight junctions, and synthetize N-Cadherin, which provides a mechanism for trans-endothelial migration; during MET, the process is reversed. Interestingly, SARS-CoV-2 influences these programs (Figure 6), and it seems that it blocks both EMT and MET; the repression of EMT prevents dendritic cells from checking the epithelial sur-face environment with the branching projections (dendrites), and the repression of MET serves to achieve a better possibility of crossing the epithelial barrier (the virus is smaller than a dendrite).

In the case of EMT, the S protein post-transcriptionally represses ZEB2 (S1C), VEZF1 (S2), and PEG10 (S2), and the N protein represses FOXC1. ZEB2 (zinc finger E-box binding homeobox 2) represses the transcription of E-cadherin and miR200, and activates N-cadherin and metastasis/invasion (Fardi et al., 2019). VEZF1 (vascular endothelial zinc finger 1) is a transcription factor that activates vasculogenesis and angiogenesis (Xiong et al., 1999). It represses the expression of the antiangiogenic factor CITED2 in endothelial cells (AlAbdi et al., 2018) and regulates the cardiac structure and contractile function (Paavola et al., 2020). PEG10 (paternally expressed gene 10), which is derived from the Ty3/Gypsy retrotransposon family, plays an important role in placenta formation and adipocyte differentiation (Xie, et al., 2018). It is overexpressed in several cancers, especially in HCC, where it activates EMT, decreases the expression levels of E-cadherin and increases the expression levels of the mesenchymal marker vimentin (Xie, et al., 2018). FOXC1 (fork-head box C1) is a forkhead family transcription factor that plays essential roles in mesenchymal lineage specification and organ development during normal embryogenesis (Niall Gilding and Somervaille, 2019). FOXC1 activates genes heralding EMT and tumor migration, it is a positive regulator of cancer stem cell function, and appears to be a downstream target of HIF1α-signaling, i.e., the FOXC1 expression enhances the adaptation to tumor hypoxia (Niall Gilding and Somervaille, 2019). In human bronchial epithelial cells (16HBE) transfected with FOXC1-siRNA, fibrosis, apoptosis, inflammatory cytokines and oxidative stress were detected (Xia et al., 2019). In addition to the mentioned repressions, the M protein promotes TAGLN/SM22 (transgelin), which stabilizes actin gels *in vitro* and may also be involved in cell migration; it suppresses the expression of the matrix metalloproteinase 9 (MMP9), which suppresses tissue remodeling and EMT (Assinder et al., 2009). In congenital heart disease pulmonary arterial hypertension (CHD-PAH), transgelin is significantly upregulated in the lung tissue (Huang et al., 2018). Interestingly, transgelin was found among the cellular proteins in influenza virus particles (Shaw et al., 2008).

In the case of MET, the S protein post-transcriptionally represses TJP2 and the N protein represses ARHGAP9 and RASIP1. TJP2/ZO2 (tight junction protein 2) is a protein with a scaffold function in the polymerization of claudins (González-Mariscal et al., 2019). In cytoplasm, it associates with the actomyosin of cytoskeleton and with junctional proteins; in the nucleus, it works as a repressor that is involved in the transcription of genes, which leads to cell undifferentiation and proliferation (González-Mariscal et al., 2019). ARHGAP9 (RHO GTPase activating protein 9) is a GAP for RHOA, CDC42 and RAC1, which are important in controlling cell morphology and motility in response to extracellular stimuli, thus organizing the actin cytoskeleton (Furukawa et al., 2001). In HCC (hepatocellular carcinoma), ARHGAP9 supported MET and E-cadherin upregulation, and inhibited migration (Zhang et al., 2018). RASIP1 (RAS interacting protein 1) works like an endothelial specific effector for RAP1 and RAS, and is important for angiogenesis and vascular development (Gingras et al., 2016). RASIP1 regulates cell junctions and cytoskeleton organization by regulation of CDC42 activity, which is required for lymphatic lumen maintenance (Liu et al., 2018). In the tubulogenesis of blood vessels, RASIP1-mediated promotion of CDC42 activity is critical for blood vessel lumen opening and expansion (Barry et al., 2016).

One of the most important findings in the SARS-CoV-2 protein interaction map is that SARS-CoV-2 hijacks and use the cullin-2 (CUL2) E3 ubiquitination pathway, ORF10 interacts with members of a RING E3 ligase complex, specifically with the CUL2-ZYG11B complex, where ZYG11B is the highest scoring protein (Gordon et al., 2020). Using the method described here, a sequence for the post-transcriptional promotions of ZYG11B was identified in the S1A-domain and the sequence for the promotions of DCUN1D5 was identified in the S2-subunit (Figure 2). ZYG11B (zyg-11 family member B) is a substrate adaptor in the CUL2 E3 ligase complex and targets N-terminal glycine degrons for proteasomal degradation (N-degron pathway). N-terminal glycine degrons are strongly enriched at caspase cleavage sites or are exposed after failure of N-myristoylation (Eldeeb et al., 2019). DCUN1D5/SCCRO5 (defective in cullin neddylation 1 domain containing 5) is a key component for neddylation of the CUL2 E3 ligase complex, and neddylation is a major regulator of ubiquitin–proteasome pathway activity; for example, NEDD8 (N8) increases the activity of ECV (an E3 ubiquitin ligase complex) to HIF1α (Sufan and Ohh, 2006). DCUN1D5/SCCRO5 is also a putative therapeutic target in oral and lung squamous cell carcinoma (Bommeljé et al., 2014).

Angiogenesis and metastasis are the major causes of tumor progression, and during tumor progression, integrins interact with extracellular matrix molecules such as vitronectin, collagen, or glycolipids to trigger signal the transduction involved in cell migration and tumor metastasis, and high integrin αV expression is promoted by BRD1-mediated acetylation (Cai et al., 2018). Interestingly, bromodomain containing 1 (BRD1) is a protein that is post-transcriptionally repressed by SARS-CoV-2 N protein (Figure 3B). BRD1 forms a complex with HBO1-histone acetyltransferase, and the HAT complex is responsible for global acetylation of H3K14 and is required for fetal liver erythropoiesis (Mishima et al., 2011). Interestingly, erythropoietin could be a candidate for the supportive treatment of COVID-19 (Hadadi et al., 2020). In monocyte-derived macrophages, silencing of BRD1 decreased the LPS-induced expression of TNFα, but did not significantly affect the expression of IL6 and IL8 (Klein et al., 2018). In synovial fibroblasts, silencing of BRD1 decreased the basal expression of MMP1 (matrix metallopro-teinase-1), but not LPS-induced MMP3, IL6 and IL8 (Klein et al., 2018).

The M protein post-transcriptionally represses SGCG and TOR1AIP1, which are the proteins that may be connected to respiratory difficulties (Figure 3A). SGCG (sarcoglycan gamma) is subunit of the sarcoglycan complex that is formed together with α-, β-, and δ-sarcoglycan subunits. Sarcoglycan subcomplex is an important part of the dystrophin-associated glycoprotein complex, which is a main connector between the extracellular matrix and the subsarcolemmal cytoskeleton (Israeli et al., 2019). The sarcolemma is protected against muscle contraction-induced damage by the sarcoglycan complex, and the loss of this protection leads to the sarcoglycanopathies (α-, β-, γ-, and δ-types). Sarcoglycanopathies are necrotic processes that lead to progressive muscle wasting (Israeli et al., 2019). The respiratory muscles, particularly the diaphragm, can be affected by the dystrophic process, which may result in a restrictive lung disease and secondary to cardiomyopathy (Hack et al., 1998; Roberts et al., 2015). TOR1AIP1/LAP1 (torsin 1A interacting protein 1 or lamina-associated polypeptide 1) is a regulatory cofactor of torsins located in the inner nuclear membrane (Rampello et al., 2020). These torsins preserve the nuclear envelope membrane integrity and require direct contact with TOR1AIP1/LAP1; mutations affecting this direct contact result in dystonia, muscular dystrophy, cardiomyopathy, and deafness (Rampello et al., 2020). Respiratory disorders associated with dystonia can lead to upper airway dysfunction and chest and diaphragmatic dysfunction (Mehanna and Jankovic, 2012).

The capsid protein N represses the collagen type XIX alpha 1 chain. COL19A1 is a nonfibrillar homotrimer collagen that belongs to the FACIT-family (fibril-associated collagens with interrupted triple helices; Oudart et al., 2017). In mouse skeletal muscle development, the various COL19A1 levels regulate muscle differentiation. In the hippocampus of adult mice, the repression of this collagen may contribute to complex brain disorders (Calvo et al., 2020). In the first 3 wk after the double inactivation of the COL19A1 gene, the mice had a motor dysfunction in smooth muscles and mortality was greater than 95% (Oudart et al., 2017). In adult humans, type XIX collagen tissue distribution is targeted to specific areas, mainly vascular, neuronal and epithelial basement membranes (Oudart et al., 2017), as well as to lymph nodes and the spleen.

The M protein downregulates NHLRC3 (NHL repeat containing 3), which is a mammalian structural analogue of honey bee royalactin (Wan et al., 2018). Royalactin was identified as a potent activator of a pluripotency gene network through the modulation of chromatin accessibility, which maintains mouse embryonic stem cell self-renewal (Wan et al., 2018). Royalactin cultured cells also occupy a more naive ground state that are capable of generating chimeric animals with germline transmission (Wan et al., 2018). The spontaneous fusion of cells might be required for development and tissue repair, and there is no doubt that cell fusion contributes to the regeneration of liver tissue; however, it is not without risks, e.g., cell fusion may explain the genesis of cancer or the emergence of new viruses through recombination (Ogle et al., 2005). Interestingly, the N protein post-transcriptionally activates GCM1 (glial cells missing homologue 1), which is a critical factor in controlling placental cell fusion. GCM1 activates fusogenic glycoproteins syncytin 1 and 2 (retroviral loci ERVWE1 and ERVFRDE1), and fusogenic receptor MFSD2A (Yu et al., 2002; Liang et al., 2010; Matsuura et al., 2011). GCM1 also upregulates the placental growth factor (PGF), which is a member of the VEGF (vascular endothelial growth factor) family and is responsible for angiogenesis in human placenta (Chiu et al., 2018).

### Altering Lipid Homeostasis as a Consequence of SARS-CoV-2 Infection

**FIGURE 7:**
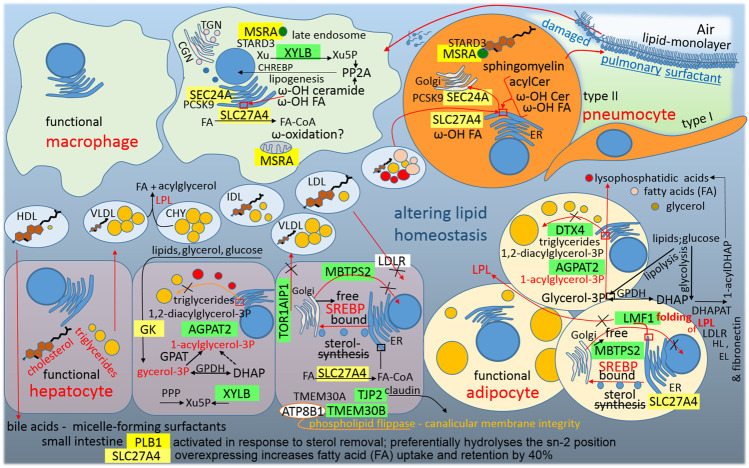
A graphic illustration of “Altering lipid homeostasis as a consequence of SARS-CoV-2 infection.” Green highlights show the alignments with the complement sequence (post-transcriptionally repressed); yellow highlights show the alignments with the reverse complement sequence (post-transcriptionally promoted). The functions of the identified genes/proteins by the methodology are summarized in Supplementary Table S1.

By attacking host-lipid biogenesis pathways, viral infections modulate lipid levels in blood plasma, which has a crucial role in guiding the propagation of the enveloped viruses and in altering immune responses (De Silva et al., 2020) (Figure 7). Surprisingly, the S2 subunit provides a sequence (residues 749–782) for post-transcriptional repression of MBTPS2/S2P (membrane bound transcription factor peptidase site 2). In mammalian cells, S2P is essential for the activation of SREBPs (sterol regulatory element binding proteins), which that activate genes encoding enzymes of cholesterol and fatty acid biosynthesis; in the absence of S2P, Chinese hamster ovary cells require exogenous cholesterol to survive (Rawson, 2013). The repression of cholesterol synthesis is unexpected because membrane cholesterol positively modulates the oligomeric status and peptide-membrane interaction of the fusion peptide (S2, residues 770–788) of SARS-CoV, which is crucial for virus entry into the host cell (Meher et al., 2019). Nevertheless, sharp decreases of TC, HD and LDL-cholesterol were observed in blood plasma at the initial stage of COVID-19 infection (Hu et al., 2020; Sorokin et al., 2020; Wu et al., 2020). The question is why S2 sequence (749–782) for post-transcriptional repression of MBTPS2/S2P is so close to the fusion peptide of S2 glycoprotein (SARS-CoV, residues 770–788).

The S1C domain promotes MSRA (methionine sulfoxide reductase A), which is essential for the protection of proteins against oxidative stress. The myristoylated form of MSRA is a late endosomal protein that may protect endosomal proteins from oxidative damage and may play a role in lipid metabolism because STARD3 (StAR-related lipid transfer domain containing 3) is its binding partner, and STARD3 mediates the transport of cholesterol from ER to the endosome (Lim et al., 2018). Additionally, the N protein promotes SEC24A, which is the coat protein complex II (COPII) component that is responsible for the selection of specific cargo, for example PCSK9, for packaging into ER-Golgi transport vesicles (Brandizzi and Barlowe, 2013). Proprotein convertase subtilisin/kexin type 9 is a secreted and circulating factor that negatively regulates cell surface LDL receptor (LDLR) expression (Chen et al., 2013). LDLR, in the liver and other cell membranes, initiates the ingestion of LDL-particles from extracellular fluid into cells. Taken together, it seems that the virus blocks cholesterol synthesis (lowered MBTPS2/S2P) and blocks LDL-cholesterol ingestion (increased PCSK9); however, the virus supports the intracellular transport of cholesterol from the ER to the endosome.

The linker sequence between S1A and S1B domains promotes phospholipase B1 (PLB1). As was already mentioned above, PLB1 is a sperm phospholipase that is activated in response to sterol removal and is released into the extracellular fluid by proteolytic cleavage. The resultant active PLB fragment can stimulate the initiation of acrosome exocytosis and membrane fusion (Asano et al., 2013). PLB1 can hydrolyze both sn-1 and sn-2 acyl chains in phospholipid substrates, thus displaying phospholipase and lyso-phospholipase activities, and therefore plays an important role in the epidermal barrier function (Maury et al., 2002). Actually, the PLB1 precursor was purified from normal human granulocytes, and after activation, the enzyme showed deacylation activity against phospholipids, including phosphatidylcholine (PC), phosphatidylinositol, phosphatidylethanolamine and lysophospholipids (Xu et al., 2009). Interestingly, PLB1 preferentially hydrolyses sn-2 acyl chains, and LPC (12:0/0:0) is upregulated in the COVID-19 signatures in the plasma lipidome (De Silva et al., 2020; Wu et al., 2020). The next question is whether the virus lowers cholesterol because of the use of this enzyme.

The S1A-domain represses XYLB, xylulokinase, which is active in the liver as the final enzyme in the glucuronate-xylulose pathway. Its product, xylulose 5-phosphate (Xu5P), is linked to the pentose-phosphate pathway and represents a key metabolic regulator (glucose-lipogenesis), (Bunker et al., 2013). Xu5P activates protein phosphatase 2A (PP2A), dephosphorylating of CHREBP promotes its nuclear localization and DNA binding. In the nucleus, active transcription factor CHREBP activates genes of enzymes that transform the end-products of glycolysis to the synthesis of lipids (Uyeda and Repa, 2006). During SARS-CoV-2 infection, Xu5P decreases sharply in the plasma and was therefore proposed as one of the potential metabolomics biomarkers of COVID-19 (De Silva et al., 2020; Wu et al., 2020).

The N protein represses AGPAT2, DTX4 and LMF1 and promotes SLC27A4/FATP4. AGPAT2 (1-acylglycerol-3-phosphate O-acyltransferase 2) is abundant in fatty tissue and catalyzes the second step in the triglyceride synthesis from glycerol 3-phosphate (Vegiopoulos et al., 2017). Functional adipocytes metabolize and store excess circulating lipids and glucose in the inert form of triglycerides in the lipid droplets; impairment of triglyceride storage, for example by mutations in AGPAT2, increases the flux of fatty acids away from adipocytes and accumulates the intermediate pathway LPA (lysophosphatidic acids), (Vegiopoulos et al., 2017). Increased LPA levels may further decrease adipose tissue mass and functionality by inhibiting adipogenesis (Vegiopoulos et al., 2017). Interestingly, LPA-(18:1/0:0) increases markedly in the COVID-19 plasma lipidome (Wu et al., 2020). DTX4 (deltex E3 ubiquitin ligase 4) polyubiquitinates K48 of TBK1 and mediates its degradation (Cui et al., 2012). As was already mentioned above, TBK1 kinase activates type I interferon signaling and NLR4 (NOD-like receptor 4) uses DTX4 for the negative regulation of type I IFN-signaling, because aberrant production of type I IFN can have an autoimmune pathological role (Cui et al., 2012). In 3T3-L1 preadipocytes, DTX4-knockdown reduced the number of lipid droplets and inhibited adipogenesis (Wang et al., 2017). Taken together, downregulation of AGPAT2 and DTX4 supports the accumulation of LPA and free fatty acids (FAs) and alters the excess lipid storage in the inert form of triglycerides in the cellular lipid droplets (Figure 7), which can be seen in the COVID-19 plasma lipidome (Wu et al., 2020). The repressed LMF1 (lipase maturation factor) is required for proper lipoprotein lipase (LPL) folding and its exit from the ER. LPL is a secreted lipase that clears triglycerides from the blood (Roberts et al., 2018). Down-regulated LPL may be a reason for the observed accumulation of triglycerides in the plasma during COVID-19 infection (Wu et al., 2020). The promoted SLC27A4/FATP4 (solute carrier family 27 member 4 or fatty acid transport protein 4) is located on the ER, mainly in the liver, and overexpressing FATP4 increases uptake and retention of FAs by 40% (Grevengoed et al., 2014). SLC27A4/FATP4 exhibits acyl-CoA synthetase activity and activates mainly ω-hydroxy fatty acids (ω-OH FA; Yamamoto et al., 2019) in the acylceramide synthetic pathway (Hirabayashi et al., 2019). Ceramide intermediate, a fusion product of dihydrosphingosine and the activated ω-OH FA, has been demonstrated to be a powerful tumor suppressor and can support type 2 immune responses against the tissue (Figure 5). In general, beyond the induction of the apoptosis of some exhausted macrophages, ceramide acts to regulate monocyte/macrophage functions, including killing capacity and cytokine production (Bai and Guo, 2018; Li et al., 2018). The ceramide accumulation in the plasma is observed during COVID-19 infection (Wu et al., 2020). Sphingomyelin synthase (phosphatidylcholine:ceramide cholinephosphotransferase) transfers a phosphocholine headgroup from phosphatidylcholine (PC) to ceramide, yielding sphingomyelin (SM) and releasing diacylglycerol (DG), (Agassandian and Mallampalli, 2013). SM is a pulmonary surfactant component for which levels associated with the surfactant in alveolar lavage are typically low, but increase in acute or chronic lung injury (Agassandian and Mallampalli, 2013). Interestingly, the accumulation of DG and decrease of PC are observed in the plasma during COVID-19 infection (Wu et al., 2020), (Figure 7).

In the plasma during COVID-19 infection, glycerol 3-phosphate is downregulated and its oxidized form (glycerol 3-phosphate +2H) is upregulated (Wu et al., 2020; De Silva et al., 2020). This is interesting because a glycerol kinase (GK) promotive sequence was found in the N protein (Figure 3B). The downregulation of glycerol 3-phosphate can be explained by the upregulation of its oxidized form, or by the fact that glycerol 3-phosphate is a central metabolite and the major source of carbon and energy for *Mycoplasma pneumoniae* (Blötz and Stülke, 2017), which is the most common non-viral pathogen detected in Qingdao COVID-19 patients (Tang et al., 2021).

The abovementioned repressed TOR1AIP1/LAP1 (Figure 3A) makes a torsin-LAP1 complex that contributes to nuclear pore complex biogenesis (Ungricht and Kutay, 2017) and cellular lipid metabolism (Rampello et al., 2020). Mutations affecting the LAP1 or TOR1A interaction result in dystonia, muscular dystrophy, cardiomyopathy, and deafness (Rampello et al., 2020). When mouse livers are conditionally depleted of TorsinA or Lap1, hepatocytes exhibit a decrease in the triglyceride secretion rate and plasma cholesterol concentration (Rampello et al., 2020). It seems that the virus inhibits hepatic triglyceride synthesis (DTX4 and AGPAT2) and secretion (TOR1AIP1/LAP1), but supports the blood level of dietary triglycerides (LMF1, inhibited folding of LPL, chylomicrons).

The S2-subunit post-transcriptionally represses TMEM30B/CDC50B. Transmembrane protein 30B is a CDC50–accessory β-subunit of a P4-ATPase complex (α/β), (Andersen et al., 2016). Phospholipid flippases of the type IV P-type ATPase family (P4-ATPases) are essential components of the Golgi, plasma membrane and endosomal system that actively transport or flip phospholipids across cell membranes (Andersen et al., 2016). TMEM30B/CDC50B associates with α-subunit ATP8B1, which preferentially localizes to the plasma membrane of the cells where the complex transports specific phospholipids from the extracellular to the cytoplasmic leaflet (Andersen et al., 2016). Mutations in ATP8B1 are responsible for liver cholestasis, and affected individuals are more susceptible to pneumonia (Andersen et al., 2016).

## Conclusions

In the process of SARS-CoV-2 pathogenesis (COVID-19), it is clear that the virus alters immune homeostasis, the pulmonary epithelial tissue homeostasis and the lipid homeostasis in the human body; however, little is known about the genes that are involved with and are influenced by the virus. The theoretical protein–RNA recognition code was used as a tool for the search for the human transcriptome that identified such genes/proteins. Despite the theoretical level, it can be concluded that the genes can be logically connected (Supplementary Table S1). It is very interesting and supportive that compatible sequences, which repress host genes, were located in the intrinsically disordered region of the nucleocapsid protein. In this regard, intrinsically disordered regions of the SARS-CoV-2 proteome, especially the N-protein inter-domain linker region, were identified as harboring active mutations that accumulate during the first wave of the pandemic (Tomaszewski et al., 2020).

In the alteration of immune homeostasis (Supplementary Table S1), it can be seen that the virus supports IL1α/β–IL1R1 signaling (IL1RAP coreceptor, N protein), this signaling further leads to the expression of cytokines and chemokines, including IL1α/β, and the initial round of IL1α/β–IL1R1 signaling can lead to self-perpetuating inflammation–inflammatory storm (Malik and Kanneganti, 2018). The virus also supports type 2 immunity (immunity defense against helminths and venoms, IL4 cytokine, allergy/asthma; Annunziato et al., 2015); the virus represses DENND1B, INHBA, KHSRP, and YTHDF1, which represent the second circle that can lead to self-perpetuating inflammation–inflammatory storm. The second circle accelerates TH2-driven immune responses. However, to guard the infected cells, the virus promotes MUC16 on the surface of infected cells, which interacts with the inhibitory receptor SIGLEG9 on the surface of NK, B cells, and monocytes (Supplementary Table S1). On the surface of plasmacytoid dendritic cells, the virus supports a specific inhibitory receptor PTPRS.

In the alteration of homeostasis in the pulmonary epithelial tissue (Supplementary Table S1), the virus blocks the function of cilia in airway multiciliated cells (RABEP1, DAW1, LRCH3, TCTN1, CCDC78, and FAM92A) and alters epithelial-mesenchymal transition (EMT) and reverse mesenchymal-epithelial transition (MET), which are the molecular programs involved in wound healing. In the case of EMT, S protein post-transcriptionally represses ZEB2, VEZF1, PEG10 and FOXC1. In the case of MET, the S protein post-transcriptionally represses TJP2, ARHGAP9 and RASIP1.

One of the most important findings in the SARS-CoV-2 protein interaction map was that SARS-CoV-2 hijacks and use the cullin-2 (CUL2) E3 ubiquitination pathway, viral ORF10 interacts with the ZYG11B substrate adaptor of CUL2 E3 complex (Gordon et al., 2020). There is a consensus, using the method described here, a sequence for promotion of ZYG11B was identified in the S1A-domain, and the sequence for promotion of DCUN1D5 was identified in the S2-subunit (Figure 2). DCUN1D5 is a key component for neddylation/activation of the CUL2 E3. Viral ORF10 uses ZYG11B-CUL2 E3 complex and S post-transcriptionally promotes ZYG11B and DCUN1D5.

Surprisingly, the S2 subunit provides a sequence for post-transcriptional repression of MBTPS2/S2P (membrane bound transcription factor peptidase site 2), which is essential for the activation of genes encoding enzymes for cholesterol biosynthesis. Nevertheless, it is in accordance with the sharp decrease of cholesterol in blood plasma observed at the initial stage of COVID-19 infection (Hu et al., 2020; Sorokin et al., 2020; Wu et al., 2020). Additionally, S1A-domain represses xylulokinase XYLB, and decreasing of its product (Xu5P) was proposed as one of the potential metabolomics biomarkers of COVID-19 (De Silva et al., 2020; Wu et al., 2020).

In the case of the SARS-CoV-2 life cycle in the host cells, the virus traffics through the endo-lysosomal system. Interestingly, the protein–RNA recognition method described here identified a compatible sequence in the S1A-domain for post-transcriptional promotion of PIKFYVE, which is one of the critical factors for SARS-CoV-2 entry into the host cell (Ou et al., 2020), and it supports lysosome fission and enables the exit from lysosomes (Saffi and Botelho, 2019). The S1C domain has a sequence for the promotion of MSRA, and its myristoylated form is localized to late endosomes and protects endosomal proteins from oxidative damage. In addition, MSRA is a binding partner of STARD3, which mediates the transport of cholesterol from the endoplasmic reticulum to the endosome. The linker sequence between S1A and S1B domains promotes phospholipase PLB1. PLB1 is activated in response to sterol removal and released into the extracellular fluid by proteolytic cleavage; after that, the enzyme has deacylation activity against phospholipids, including phosphatidylcholine, phosphatidylinositol, phosphatidylethanolamine and lysophospholipids (Xu et al., 2009). Interestingly, the lyso-intermediates, such as lysophosphatidylcholines, lysophosphatidylinositol and lysophosphatidylethanolamines, are up-regulated in the COVID-19 signatures of the plasma lipidome (De Silva et al., 2020; Wu et al., 2020).

In summary, the protein–RNA recognition code leads to protein genes relevant to the SARS-CoV-2 life cycle and pathogenesis. This gives credence to both the proposed recognition code and the methodological pipeline used to identify the genes. The identified genes/proteins regulate endo-lysosomal transport and amplify alarmin signal and type 2 immune responses. The viral protein-host RNA interactions support pro-inflammatory responses, but inhibit some other parts of immunity, inhibit formation and function of cilia, inhibit tissue wound healing (EMT and MET programs), and inhibit intracellular cholesterol in ER, but promote lysophospholipids (Supplementary Table S1).

## Data Availability

The original contributions presented in the study are included in the article/Supplementary Material, further inquiries can be directed to the corresponding authors.
