# Supplementary material for: Theoretical Analysis of S, M and N Structural Proteins by the Protein–RNA Recognition Code Leads to Genes/proteins that Are Relevant to the SARS-CoV-2 Life Cycle and Pathogenesis
Source: Front Genet. 2021 Sep 29;12:763995. doi: 10.3389/fgene.2021.763995 (PMC8511677; doi:10.3389/fgene.2021.763995)
Supplement: Supplementary file 1 [file DataSheet1.pdf]

Theoretical analysis of S, M and N structural proteins by the Protein–RNA recognition code leads to genes/proteins that are relevant to the SARS-CoV-2 life cycle and pathogenesis

Jozef Nahalka [1,2,*]

[1] Institute of Chemistry, Centre for Glycomics, Slovak Academy of Sciences, Dubravska cesta 9, SK-84538 Bratislava, Slovak Republic

[2] Institute of Chemistry, Centre of excellence for white-green biotechnology, Slovak Academy of Sciences, Trieda Andreja Hlinku 2, SK-94976 Nitra, Slovak Republic

*** Correspondence:**
Jozef Nahalka
nahalka@savba.sk

Supplementary Material 1

1 Supplementary Table

SM Table 1. Molecular pathways affected by COVID-19 infection at the level of viral protein–host RNA interactions. The results are obtained by the analysis of S, M and N structural proteins with the theoretical protein–RNA recognition code (Nahalka, 2014, 2019). In addition to viral protein–host protein and viral RNA–host protein interactions, the presented theory assumes that the virus also take control over the entire cellular system at the level of the viral protein–host RNA interactions.

	Viral protein	Host transcriptome		Pathway/function description
life cycle		**Promotion**	**Repression**	*graphically illustrated in the Figure 4*
endo-lysosomal transport	S1A	PIKFYVE		SARS-CoV-2 entry; phosphorylates PI(3)P to PI(3,5)P2; early endosome maturation to the late endosomes; supports lysosome fission and enables the exit from lysosomes
	S1A		RABEP1	early endosome associated activator for RAB5, which positively regulates PI(3)P synthesis
	S2	AP5M1		the μ subunit in AP5; transport from late endosomes to the Golgi
	S2		DENND1B	GEF for RAB35 and AP2; endocytic recycling, RAB35 activates PI3K-AKT pathway

	S1C	MSRA		protects endosomal proteins from oxidative damage
	S2	ASTN2		endo-lysosomal protein, which is involved in trafficking and degradation of surface proteins
	N		KHSRP	negatively regulates cap-independent translation processes
	N	SLC27A4		an acyl-CoA synthetase that activates FAs and channels them towards oxidation
	N	SEC24		COPII-component, which mediates transport from the ER to the Golgi
	S1A	PLB1		stimulates of sperm acrosome exocytosis and membrane fusion; activated in response to the cholesterol decrease
	S2		TMEM 30B	the β-subunit of the phospholipid flippase, which associates with the α-subunit ATP8B1
immune homeostasis				*graphically illustrated in the Figure 5*
alarmin signal amplifica-tion	N	IL1RAP		IL1α–IL1R1-IL1RAP and IL33–IL1R4-IL1RAP signaling; IL1α and IL33 are alarmins that act in a similar way as DAMP molecules
amplifica-tion of type 2 immune responses	N		YTHDF1	m6A-modification RNA reader; its loss in classical dendritic cells activates T_H2 cells
	N		KHSRP	a negative regulator of IL4 expression (T_H2 cells)
	S2		DENND1B	important for down-modulation of the T cell receptor in T_H2 cells, its loss increases production of IL4, IL5
	S2		INHBA	activates T_{reg} and inhibits T_H2 cell responses

inhibition of immunity	M	ARHGAP 19		a GAP for RHOA; RHOA is involved in dendritic cell and macrophage migration, and is the key switch of innate and adaptive immunity
	S2	PTPRS		inhibitory receptor on human plasmacytoid dendritic cells; the ligands likely involve diverse heparan sulfates
	S1C		ZEB2	loss of ZEB2 lower macrophage populations, critical for maintaining the tissue identities of macrophages
	S2		FUT9	macrophage adhesion to the epithelial surface
	N		ARHGAP9	a GAP for CDC42 and RAC1; controlling cell motility and morphology
	S1A	PIKFYVE		enables exit from lysosomes; supports IL12 and IL23 expression (T_H1 and T_H17 activation); preventing infiltration of eosinophils and lymphoid cells
pro-inflammato-ry	N		DTX4	the negative regulator of type I IFN signaling and antiviral immunity
	N	TNFRSF12 A		TNF receptor; persistent activation can lead to chronic inflammation, stimulates bronchial epithelial cells to produce IL8 and GM-CSF
			CEACAM-20	downregulated in inflammatory bowel disease (IBD)
	S2		NOX1	NADPH oxidase, required for wound healing, and repression of the NOX1 can lead to IBD
	N	HYAL2		hyaluronidase responsible for the continuous hyaluronan fragmentation
inhibition of immunity	S2	MUC16		transmembrane mucin that serves as a ligand for inhibitory receptor SIGLEG9, suppression of NK cells, B cells, and monocytes
	M		PCSK7	the furin-like proprotein convertase generally found in cells of the immune system

	N		DOC2A	positively regulates lung mast cell degranulation
	S1A	PARG		poly(ADP-ribose) glycohydrolase, which suppresses PAR-dependent ubiquitylation-protein degradation
homeostasis in the pulmonary epithelial tissue				*graphically illustrated in the Figure 6*
inhibition of formation and function of cilia	S1A		RABEP1	inhibition of RABEP1 causes defective ciliary function
	N		CCDC78	essential for centriole amplification in multiciliated cells
	N		TCTN1	a protein of a transition zone complex that regulates mammalian ciliogenesis
	M		FAM92A	a protein of a transition zone that plays a crucial role in the formation and function of cilia
	S1A	DAW1		an adaptor for the transport of outer dynein arms
	S2	LRCH3		displaces septins from actin filaments, septins are important in the biogenesis and function of cilia
	N	XPNPEP3		upregulation of this mitochondrial enzyme somehow negatively influence the cilia formation and function
inhibition of EMT	S1C		ZEB2	transcription factor that represses transcription of E-cadherin and MIR200, and activates N-cadherin
	S2		VEZF1	transcription factor that that activates vasculogenesis and angiogenesis
	S2		PEG10	decreases E-cadherin and increases the expression levels of the mesenchymal marker vimentin
	N		FOXC1	transcription factor with essential roles in mesenchymal lineage specification

	M	TAGLN		suppresses expression of the matrix metalloproteinase 9, which suppresses tissue remodeling and EMT
inhibition of MET	S1B		TJP2	tight junction protein with a scaffold function, a repressor of undifferentiating and proliferation genes
	N		ARHGAP9	GAP for RHOA, CDC42 and RAC1; ARHGAP9 supports MET and E-cadherin upregulation
	N		RASIP1	regulates cell junctions; effector for RAP1 and RAS and is important for angiogenesis and vascular development
	S1A	ZYG11B		SARS-CoV-2 hijacks the CUL2 E3 ubiquitination pathway, ORF10 interacts with CUL2-ZYG11B complex, where ZYG11B is a substrate adaptor
	S2	DCUN1D5		neddylation of the CUL2 E3, a positive regulator of ubiquitin–proteasome pathway activity
	N		BRD1	forms a complex with HBO1-histone acetyltransferase; HAT complex is required for fetal liver erythropoiesis; in macrophages, silencing of BRD1 decreases the TNFα, but did not significantly affect IL6 and IL8
	M		SGCG	subunit of the sarcoglycan complex, which protects the sarcolemma against muscle contraction-induced damage
	M		TOR1AIP1	a positive regulatory cofactor of torsins, torsins preserve the nuclear envelope-membrane integrity
	N		COL19 A1	collagen that is targeted to vascular, neuronal and epithelial basement membranes
	M		NHLRC3	a mammalian structural analogue of honey bee royalactin

	N	GCM1		cell fusion; it activates fusogenic glycoproteins syncytin 1,2 (retroviral loci), and fusogenic receptor MFSD2A
lipid homeostasis				*graphically illustrated in the Figure 7*
inhibition of intracellular cholesterol in ER	S2		MBTPS2	peptidase essential for activation of SREBPs that activate genes for cholesterol and fatty acid biosynthesis
	S1C	MSRA		endosomal protein that binds STARD3; STARD3 mediates transport of cholesterol from ER to the endosome
	N	SEC24A		COPII-component responsible for the selection of specific cargo (PCSK9), PCSK9 negatively regulates cell surface LDL receptor
	S1A	PLB1		phospholipase that is activated in response to cholesterol removal
inhibition of intracellular triglyceride synthesis in ER	S1A		XYLB	xylulokinase, its product Xu5P decreases sharply in the plasma during SARS-CoV-2 infection; Xu5P activates PP2A-CHREBP pathway that transform the glycolysis to the synthesis of triglycerides
	N		AGPAT2	catalyzes the second step in triglyceride synthesis from glycerol 3-phosphate; its substrate, lysophosphatidic acid (18:1/0:0), increases markedly in the COVID-19 plasma
	N		DTX4	in preadipocytes, DTX4-knockdown reduces the number of lipid droplets (triglycerides)
	N		LMF1	important factor for lipoprotein lipase exit from ER; LPL degrades triglycerides; as a consequence LPL stays and degrades triglycerides in ER
	N	SLC27A4		fatty acid transport protein 4, increases uptake and retention of FAs in ER; channels FAs towards oxidation; exhibits acyl-CoA synthetase activity towards ω-OH FAs

promotion of intracellular lysophospholipids	N	GK		glycerol kinase; the lysophospholipids are upregulated in the plasma during COVID-19 infection
	M		TOR1AIP1	when mouse livers are conditionally depleted of TOR1AIP1, hepatocytes exhibit a decrease in the triglyceride secretion and plasma cholesterol
	S2		TMEM 30B	one type of β-subunit of a P4-ATPase complex (α/β); associates with α-subunit ATP8B1; α/β-phospholipid flippase flips phospholipids across cell membrane

2 Supplementary sequence information

S protein, blastn for surface glycoprotein and hsa transcripts

>QHD43416.1 surface glycoprotein [Severe acute respiratory syndrome coronavirus 2] **AA-sequence**

S1

```
MFVFLVLLPLVSSQCVNLTTRTQLPPAYTNSFTRGVYYPDKVFRSSVLHSTQDLFLPFFSNVTWFHAIHVSGTNGTKRFDN
PVLPFNDGVYFASTEKSNIIRGWIFGTTLDSKTQSLLIVNNATNVVIKVCEFQFCNDPFLGVYYHKNNKSWMESEFRVYSS
ANNCTFEYVSQPFLMDLEGKQGNFKNLREFVFKNIDGYFKIYSKHTPINLVRDLPQGFSALEPLVDLPIGINITRFQTLLA
LHRSYLTPGDSSSGWTAGAAAYYVGYLQPRTFLLKYNENGTITDAVDCALDPLSETKCTLKSFTVEKGIYQTSNFRVQPTE
SIVRFPNITNLCPFGEVFNATRFASVYAWNRKRISNCVADYSVLYNSASFSTFKCYGVSPTKLNDLCFTNVYADSFVIRGD
EVRQIAPGQTGKIADYNYKLPDDFTGCVIAWNSNNLDSKVGGNYNYLYRLFRKSNLKPFERDISTEIYQAGSTPCNGVEGF
NCYFPLQSYGFQPTNGVGYQPYRVVVLSFELLHAPATVCGPKKSTNLVKNKCVNFNFNGLTGTGVLTESNKKFLPFQQFGR
DIADTTDAVRDPQTLEILDITPCSFGGVSVITPGTNTSNQVAVLYQDVNCTEVPVAIHADQLTPTWRVYSTGSNVFQTRAG
CLIGAEHVNNSYECDIPIGAGICASYQTQTNSPRRAR
```

S2

```
SVASQSIIAYTMSLGAENSVAYSNNSIAIPTNFTISVTTEILPVSMTKTSVDCTMYICGDSTECSNLLLQYGSFCTQLNRA
LTGIAVEQDKNTQEVFAQVKQIYKTPPIKDFGGFNFSQILPDPSKPSKRSFIEDLLFNKVTLADAGFIKQYGDCLGDIAAR
DLICAQKFNGLTVLPPLLTDEMIAQYTSALLAGTITSGWTFGAGAALQIPFAMQMAYRFNGIGVTQNVLYENQKLIANQFN
SAIGKIQDSLSSTASALGKLQDVVNQNAQALNTLVKQLSSNFGAISSVLNDILSRLDKVEAEVQIDRLITGRLQSLQTYVT
QQLIRAAEIRASANLAATKMSECVLGQSKRVDFCGKGYHLMSFPQSAPHGVVFLHVTYVPAQEKNFTTAPAICHDGKAHFP
REGVFVSNGTHWFVTQRNFYEPQIITTDNTFVSGNCDVVIGIVNNTVYDPLQPELDSFKEELDKYFKNHTSPDVDLGDISG
INASVVNIQKEIDRLNEVAKNLNESLIDLQELGKYEQYIKWPWYIWLGFIAGLIAIVMVTIMLCCMTSCCSCLKGCCSCGS
CCKFDEDDSEPVLKGVKLHYT
```

AA-sequences are transcribed to the imaginary nucleotide sequences by 1-letter code, and then, the sequences are used for blastn-search in the human transcriptome (the conditions for the blastn-search are described in the paper):

* S1c transcript, S (Ser) transcription to C (cytidine)

```
UUUUUUUUCUUCCAGUAUCCGCAUCCCACACUCGGUAACAAUUGCCUUACCAAUUUCUUCAUCGUACUAUCGCAGCAGUAA
CUUCUAAGUAUCCCAACAUUGGGUUGCCUACACACUUUUAACCAUUUAUGAUAUGAACUUGUAAAAAAACGUACAUGUACC
CAAGCUAAUCACUUUAUAGAAGAUAAUGAUUUAAUAGAUAUACAACCUAUUGAUCAGUCCUACUUAUCUGUAUCGUACUUC
UAGCAUCCGACCCGGCCGCCCAAUGAUACGCUUUAAAAAGCUCACUAGCUACUCACAGCUACUCUAAGUAACCAUGUACCA
CUUGUCAUCAUGCUGAUUACCGUCCUACGAGAGUCAGUCAACUUAACCCUCCUAGAGUCCCAUAAUGUCAUACACUUUGGA
AUGAUCCGACGAUCAAAAAUCAAUCGGUUCGACAAUACAUGGAAAAUAGUUGACAUACUAGAUCCAUAACGCCCGAGUAGU
AGAUCUACAGUACCAGUGAACAGUUUUCUAUUACCCCUGGCAACCAUUAAAGUAUAUAGUCGCGUUCACAAAUUCUAAUGG
AUCACCACUGACACUAUUAUCCGCUGGUCUUCCGCACCAAUCUUAAAUAGCAUCUCUACAAUCCCGGUACCGCAUUACGCG
GUUGCAAUAACAAGAUCUGCGUGCCAACACACCGGCG
```

>NM_003631.4 Homo sapiens poly(ADP-ribose) glycohydrolase (**PARG**), transcript variant 1, mRNA

```
Query  55    TTCTTCATCGTACTATCGCAGCAGTAACTTCTAAGTATC-CCAACATTGGGTTGCCT  110
             |||||||| ||| ||||| ||||  | ||| ||   ||| |||||| || ||| |||
Sbjct  3119  TTCTTCATTGTAGTATCGTAGCAACAGCTTATACACATCTCCAACAGTGAGTTTCCT 3063
```

>NM_178821.2:1046-1072 Homo sapiens dynein assembly factor with WD repeats 1 (**DAW1**), transcript variant 1, mRNA

```
Query  6     tttCTTCCAGTATCCGCATCCCACACT  32
             || ||||||||||| || |||||||||
Sbjct  1072  TTCCTTCCAGTATCAGCGTCCCACACT  1046
```

>NM_024646.2:4541-4574 Homo sapiens zyg-11 family member B, cell cycle regulator (**ZYG11B**), mRNA

```
Query  113   ACACTTTTAACCATTTATGATATGAACTTGTaaa  146
             ||||||||| |||||| ||| |  || |||||||
Sbjct  4574  ACACTTTTAGCCATTTCTGAAAATAATTTGTAAA  4541
```

* S1cr transcript, S (Ser) transcription to C; transcription of reversed protein sequence

```
GCGGCCACACAACCGUGCGUCUAGAACAAUAACGUUGGCGCAUUACGCCAUGGCCCUAACAUCUCUACGAUAAAUUCUAAC
CACGCCUUCUGGUCGCCUAUUAUCACAGUCACCACUAGGUAAUCUUAAACACUUGCGCUGAUAUAUGAAAUUACCAACGGU
CCCCAUUAUCUUUUGACAAGUGACCAUGACAUCUAGAUGAUGAGCCCGCAAUACCUAGAUCAUACAGUUGAUAAAAGGUAC
AUAACAGCUUGGCUAACUAAAAACUAGCAGCCUAGUAAGGUUUCACAUACUGUAAUACCCUGAGAUCCUCCCAAUUCAACU
GACUGAGAGCAUCCUGCCAUUAGUCGUACUACUGUUCACCAUGUACCAAUGAAUCUCAUCGACACUCAUCGAUCACUCGAA
AAAUUUCGCAUAGUAACCCGCCGGCCCAGCCUACGAUCUUCAUGCUAUGUCUAUUCAUCCUGACUAGUUAUCCAACAUAUA
GAUAAUUUAGUAAUAGAAGAUAUUUCACUAAUCGAACCCAUGUACAUGCAAAAAAAUGUUCAAGUAUAGUAUUUACCAAUU
UUCACACAUCCGUUGGGUUACAACCCUAUGAAUCUUCAAUGACGACGCUAUCAUGCUACUUCUUUAACCAUUCCGUUAACA
AUGGCUCACACCCUACGCCUAUGACCUUCUUUUUUUU
```

>NM_001135671.2:304-336 Homo sapiens methionine sulfoxide reductase A (**MSRA**), transcript variant 3, mRNA

```
Query  529  TACATGCaaaaaaaTGTTCAAGTATAGTATTTACCAA  565
            |||||||||||||    |||| |||||||||| ||||
Sbjct  336  TACATGCAAAAAA----TCAAATATAGTATTTCCCAA  304
```

>XM_011532610.2:623-644 PREDICTED: Homo sapiens phospholipase B1 (**PLB1**), transcript variant X21, mRNA

```
Query  297  AATACCCTGAGATCCTCCCAAT  318
            |||||||||||| |||||||||
Sbjct  644  AATACCCTGAGAACCTCCCAAT  623
```

```
>NM_014795.3:8025-8061 Homo sapiens zinc finger E-box binding homeobox 2 (ZEB2),
transcript variant 1, mRNA
Query  507   TATTTCACTAATCGAACCCATGTACATGCaaaaaaaT  543
             ||||| ||||   ||  |||||||||||| ||||| ||
Sbjct  8025  TATTTGACTATGTGATCCCATGTACATGTAAAAACAT  8061
```

* S1g transcript, S (Ser) transcription to G (guanosine)

```
UUUUUUUUCUUGGAGUAUCCGCAUCCCACAGUCGGUAACAAUUGGGUUAGCAAUUUCUUGAUCGUACUAUGGCAGCAGUAA
CUUCUAAGUAUCGCAAGAUUGGGUUGCCUAGACAGUUUUAACCAUUUAUGAUAUGAACUUGUAAAAAAAGGUAGAUGUAGG
CAAGCUAAUGACUUUAUAGAAGAUAAUGAUUUAAUAGAUAUAGAACCUAUUGAUCAGUGCUACUUAUCUGUAUCGUACUUC
UAGGAUCCGAGGGGGCCGCCCAAUGAUACGCUUUAAAAAGCUCACUAGCUACUGACAGCUAGUCUAAGUAACGAUGUACCA
GUUGUCAUCAUGCUGAUUACCGUCGUACGAGAGUGAGUCAAGUUAAGCGUGCUAGAGUGCCAUAAUGUCAUACAGUUUGGA
AUGAUCCGACGAUCAAAAAUCAAUCGGUUCGAGAAUAGAUGGAAAAUAGUUGAGAUACUAGAUGCAUAACGGCCGAGUAGU
AGAUCUAGAGUACCAGUGAACAGUUUUGUAUUACCCCUGGCAAGCAUUAAAGUAUAUAGUCGCGUUCAGAAAUUCUAAUGG
AUCACCACUGACACUAUUAUCCGGUGGUGUUCCGCACGAAUCUUAAAUAGCAUCUCUACAAUCCCGGUAGCGGAUUACGCG
GUUGCAAUAAGAAGAUCUGCGUGCGAACACAGCGGCG
```

```
>NM_004703.5:1917-1950 Homo sapiens rabaptin, RAB GTPase binding effector protein
1 (RABEP1), transcript variant 1, mRNA
Query  160   AGGCAAGCTAATGAC--TTTATAGAAGATAATGA  191
             |||||||||||||||   ||| |||||| |||||
Sbjct  1917  AGGCAAGCTAATGACCAGTTAGAGAAGACAATGA  1950
```

```
>NM_152671.3:2001-2035 Homo sapiens phosphoinositide kinase, FYVE-type zinc
finger containing (PIKFYVE), transcript variant 3, mRNA
Query  173   ACTTTATAGAAGATAATGATTTAATAGATATAGAA  207
             || ||| |||||| | || ||||||| ||||||||
Sbjct  2035  ACATTAGAGAAGAAATTGTTTTAATAAATATAGAA  2001
```

* S1gr transcript, S (Ser) transcription to G (guanosine); transcription of reversed protein sequence

```
GCGGCGACACAAGCGUGCGUCUAGAAGAAUAACGUUGGCGCAUUAGGCGAUGGCCCUAACAUCUCUACGAUAAAUUCUAAG
CACGCCUUGUGGUGGCCUAUUAUCACAGUCACCACUAGGUAAUCUUAAAGACUUGCGCUGAUAUAUGAAAUUACGAACGGU
CCCCAUUAUGUUUUGACAAGUGACCAUGAGAUCUAGAUGAUGAGCCGGCAAUACGUAGAUCAUAGAGUUGAUAAAAGGUAG
AUAAGAGCUUGGCUAACUAAAAACUAGCAGCCUAGUAAGGUUUGACAUACUGUAAUACCGUGAGAUCGUGCGAAUUGAACU
GAGUGAGAGCAUGCUGCCAUUAGUCGUACUACUGUUGACCAUGUAGCAAUGAAUCUGAUCGACAGUCAUCGAUCACUCGAA
AAAUUUCGCAUAGUAACCCGCCGGGGGAGCCUAGGAUCUUCAUGCUAUGUCUAUUCAUCGUGACUAGUUAUCCAAGAUAUA
GAUAAUUUAGUAAUAGAAGAUAUUUCAGUAAUCGAACGGAUGUAGAUGGAAAAAAAUGUUCAAGUAUAGUAUUUACCAAUU
UUGACAGAUCCGUUGGGUUAGAACGCUAUGAAUCUUCAAUGACGACGGUAUCAUGCUAGUUCUUUAACGAUUGGGUUAACA
AUGGCUGACACCCUACGCCUAUGAGGUUCUUUUUUUU
```

```
>NM_004817.3:1518-1544 Homo sapiens tight junction protein 2 (TJP2), transcript
variant 1, mRNA
Query  495   AGTAATAGAAGATATTTCAGTAATCGA  521
             || ||||||||||||||||| ||| ||
Sbjct  1518  AGAAATAGAAGATATTTCAGAAATAGA  1544
```

```
>NM_001349180.1:875-901 Homo sapiens xylulokinase (XYLB), transcript variant 4,
mRNA
```

```
Query  102  TATCACAGTCACCACTAGGTAATCTTA  128
            ||||||  ||||||||| |||||||||
Sbjct  875  TATCACTATCACCACTATGTAATCTTA  901
```

* S2c transcript, S (Ser) transcription to C (cytidine)

CUCCACUUCACUCUGCAACUCACAACUCUCCAUCUCUCCAUUCUCUCACCUAGCUAUGGACCAGCAUUUAAGCUGCAUAGC
UCGUCUAAAAACAAUUCAUAAUAACCCUAAUGGUAUCAUUCACCACCAGCUUAAUUUAAUCUCACGUUAAAGAGUGAUCCG
AUUGCAAUAGUCUUCCUUCAAUUCAACCCUUCGCUCCGGCUGCGCCUAUCUCUAUCAGUAGUGUCAAUUAAAAAUUCAAUA
CCUGAUAACUCCCCCCUGAUAAUUAAACACUACUUAAUCCAUGCUCCUUAAUUCGUAAUACAUAUAGUUCGGUACUACAUC
AAUUGCCAUGCCCAUCCCAUCAGUUGACAGUAUGGAGAAUUCUCACCCAGUUUUAUCAUCCAAAAUCCCCCUGAAGACAUC
GAGUUUCAGCAGUUCAGAUAACAUUCCAACUUCGAGAUUUGUUAACUAACUACAUACUAAAUAAAUAAACCCAUAUGAUCG
UACCUUAUAAAUAGUAAUCAAUAACUUAUAAUGAAAAAUAGCGAUGUGUUCGUUCUUUUCUUUGGUCCGGCGUAGGGCGGC
GGAUAAAACACUUAGUAUAAC

>XM_011513242.3:1913-1941 PREDICTED: Homo sapiens leucine rich repeats and calponin homology domain containing 3 (**LRCH3**), transcript variant X13, mRNA

```
Query  448   TAACTAACTACATACTAAATAAATAAACC  476
             ||| ||| || ||| ||||||||||||||
Sbjct  1941  TAAATAAATAAATAATAAATAAATAAACC  1913
```

>NM_181718.3:112-147 Homo sapiens aspartate beta-hydroxylase domain containing 1 (**ASPHD1**), mRNA

```
Query  14   TGCAACTCACAACTCTCCATCTCTCCATTCTCTCACCT  51
            |||| ||| |||| |||| ||||||| ||||||| |||
Sbjct  147  TGCACCTC-CAACCCTCC-TCTCTCCTTTCTCTCCCCT  112
```

>NR_003255.2:893-926 Homo sapiens TSIX transcript, XIST antisense RNA (**TSIX**), antisense RNA

```
Query  495  AAATAGTAATCAATAACTTATAATGAAAAATAGC  528
            |||||| |  |||| ||| ||||||||||| |||
Sbjct  926  AAATAGGATGCAATGACTAATAATGAAAAACAGC  893
```

* S2cr transcript, S (Ser) transcription to C; transcription of reversed protein sequence

CAAUAUGAUUCACAAAAUAGGCGGCGGGAUGCGGCCUGGUUUCUUUUCUUGCUUGUGUAGCGAUAAAAAGUAAUAUUCAAU
AACUAAUGAUAAAUAUUCCAUGCUAGUAUACCCAAAUAAAUAAAUCAUACAUCAAUCAAUUGUUUAGAGCUUCAACCUUAC
AAUAGACUUGACGACUUUGAGCUACAGAAGUCCCCCUAAAACCUACUAUUUUGACCCACUCUUAAGAGGUAUGACAGUUGA
CUACCCUACCCGUACCGUUAACUACAUCAUGGCUUGAUAUACAUAAUGCUUAAUUCCUCGUACCUAAUUCAUCACAAAUUA
AUAGUCCCCCCUCAAUAGUCCAUAACUUAAAAAUUAACUGUGAUGACUAUCUCUAUCCGCGUCGGCCUCGCUUCCCAACUU
AACUUCCUUCUGAUAACGUUAGCCUAGUGAGAAAUUGCACUCUAAUUUAAUUCGACCACCACUUACUAUGGUAAUCCCAAU
AAUACUUAACAAAAAUCUGCUCGAUACGUCGAAUUUACGACCAGGUAUCGAUCCACUCUCUUACCUCUCUACCUCUCAACA
CUCAACGUCUCACUUCACCUC

>NM_032299.3:9719-9749 Homo sapiens defective in cullin neddylation 1 domain containing 5 (**DCUN1D5**), transcript variant 1, mRNA

```
Query  112   CCCAAATAAATAAATCATACATCAATCAATT  142
             |||||||||||| |||||  |||||| ||||
Sbjct  9749  CCCAAATAAATACATCATGTATCAATGAATT  9719
```

>XM_011536940.3:8774-8793 PREDICTED: Homo sapiens adaptor related protein complex 5 subunit mu 1 (**AP5M1**), transcript variant X1, mRNA

```
Query  107   GTATACCCAAATAAATAAAT  126
             ||||||||||||||||||||
Sbjct  8793  GTATACCCAAATAAATAAAT  8774
```

>NM_001080430.3:1232-1262 Homo sapiens TOX high mobility group box family member 3 (**TOX3**), transcript variant 1, mRNA

```
Query  550   CCTCTCTACCTCTCAACACTCAAC-GTCTCA  579
             ||||||| | ||||||||||| || ||||||
Sbjct  1232  CCTCTCTCCTTCTCAACACTCCACTGTCTCA  1262
```

* S2g transcript, S (Ser) transcription to G (guanosine)

```
GUCGAGUUCACUGUGCAAGUCAGAAGUCUCCAUCUGUCCAUUCUGUCACGUAGCUAUGGAGCAGGAUUUAAGGUGCAUAGC
UCGUCUAAAAACAAUUCAUAAUAACCCUAAUGGUAUGAUUCACGACGAGGUUAAUUUAAUCUCACGUUAAAGAGUGAUCCG
AUUGCAAUAGUCUUCCUUCAAUUCAACGCUUCGCUCGGGCUGCGCCUAUCUCUAUCAGUAGUGUCAAUUAAAAAUUCAAUA
GCUGAUAAGUGGCCGCUGAUAAUUAAACACUACUUAAUGGAUGCUGGUUAAUUGGUAAUACAUAUAGUUCGGUAGUACAUC
AAUUGCCAUGCGCAUCCCAUGAGUUGAGAGUAUGGAGAAUUGUCAGCCAGUUUUAUCAUCCAAAAUCCCCCUGAAGACAUC
GAGUUUGAGCAGUUCAGAUAACAUUCCAACUUGGAGAUUUGUUAACUAACUACAUAGUAAAUAAAUAAACGCAUAUGAUGG
UACGUUAUAAAUAGUAAUCAAUAAGUUAUAAUGAAAAAUAGCGAUGUGUUCGUUCUUUUCUUUGGUCGGGGGUAGGGGGGG
GGAUAAAAGACUUAGUAUAAC
```

>NM_002192.3:2526-2553 Homo sapiens inhibin subunit beta A (**INHBA**), mRNA

```
Query  542   TTTTCTTTGGTCgggggtaggggggggg  569
             ||||||||||| |||||| |||| ||||
Sbjct  2526  TTTTCTTTGGTTGGGGGTGGGGGTGGGG  2553
```

>NR_003955.1:3037-3061 Homo sapiens embigin pseudogene 1 (**EMBP1**), non-coding RNA

```
Query  218   CAGTAGTGTCAATTAAAAATTCAAT  242
             ||||| || ||||||||||||||||
Sbjct  3061  CAGTATTGCCAATTAAAAATTCAAT  3037
```

>NM_007146.2:3362-3383 Homo sapiens vascular endothelial zinc finger 1 (**VEZF1**), transcript variant 1, mRNA

```
Query  453   AACTACATAGTAAATAAATAAA  474
             |||||||||||||| |||||||
Sbjct  3362  AACTACATAGTAAAAAAATAAA  3383
```

>NM_001184961.1:6336-6372 Homo sapiens paternally expressed 10 (**PEG10**), transcript variant 1, mRNA

```
Query  229   ATTAAAAATTCAATAGCTGATAAGTGGCCGCTGATAA  265
             ||||| |||| |||||  ||| |||| || |||||||
Sbjct  6336  ATTAATAATTTAATAGAGGATGAGTGACCTCTGATAA  6372
```

>NM_001017970.2:2151-2177 Homo sapiens transmembrane protein 30B (**TMEM30B**), mRNA

```
Query  539   TTCTTTTCTTTGGTCggggtagggggg  565
             |||||||||||||||||||  || |||
Sbjct  2151  TTCTTTTCTTTGGTCGGGGAGAGTGGG  2177
```

>XM_011545678.2:1031-1064 PREDICTED: Homo sapiens monofunctional C1-tetrahydrofolate synthase, mitochondrial-like (**LOC105379443**), transcript variant X5, mRNA

```
Query  331   CATGCGCATCCCATGAGTTGAGAGTATGGAGAAT  364
             |||||||  |||||||  ||||||| || |||||
Sbjct  1031  CATGCGCTCCCCATGATATGAGAGTGTGCAGAAT  1064
```

>NR_046175.1:1427-1460 Homo sapiens methylenetetrahydrofolate dehydrogenase (NADP+ dependent) 1 like pseudogene (**LOC286297**), non-coding RNA

```
Query  331   CATGCGCATCCCATGAGTTGAGAGTATGGAGAAT  364
             |||||||  |||||||  ||||||| || |||||
Sbjct  1427  CATGCGCTCCCCATGATATGAGAGTGTGCAGAAT  1460
```

* S2gr transcript, S (Ser) transcription to G (guanosine); transcription of reversed protein sequence

CAAUAUGAUUCAGAAAAUAGGGGGGGGGAUGGGGGCUGGUUUCUUUUCUUGCUUGUGUAGCGAUAAAAAGUAAUAUUGAAU
AACUAAUGAUAAAUAUUGCAUGGUAGUAUACGCAAAUAAAUAAAUGAUACAUCAAUCAAUUGUUUAGAGGUUCAACCUUAC
AAUAGACUUGACGAGUUUGAGCUACAGAAGUCCCCCUAAAACCUACUAUUUUGACCGACUGUUAAGAGGUAUGAGAGUUGA
GUACCCUACGCGUACCGUUAACUACAUGAUGGCUUGAUAUACAUAAUGGUUAAUUGGUCGUAGGUAAUUCAUCACAAAUUA
AUAGUCGCCGGUGAAUAGUCGAUAACUUAAAAAUUAACUGUGAUGACUAUCUCUAUCCGCGUCGGGCUCGCUUCGCAACUU
AACUUCCUUCUGAUAACGUUAGCCUAGUGAGAAAUUGCACUCUAAUUUAAUUGGAGCAGCACUUAGUAUGGUAAUCCCAAU
AAUACUUAACAAAAAUCUGCUCGAUACGUGGAAUUUAGGACGAGGUAUCGAUGCACUGUCUUACCUGUCUACCUCUGAAGA
CUGAACGUGUCACUUGAGCUG

>NM_007052.4:2330-2359 Homo sapiens NADPH oxidase 1 (**NOX1**), transcript variant 1, mRNA

```
Query  63    ATAAAAAGTAATATTGAATAACTAATGATAAA  94
             ||||||| |  |||||||||| ||||||||||
Sbjct  2330  ATAAAAATT--TATTGAATAATTAATGATAAA  2359
```

>NM_006581.3:2144-2168 Homo sapiens fucosyltransferase 9 (**FUT9**), mRNA

```
Query  476   GTAATCCCAATAATACTTAACAAAA  500
             |||||||||||||||||| | ||||
Sbjct  2144  GTAATCCCAATAATACTTTAGAAAA  2168
```

>NM_024690.2:23248-23276 Homo sapiens mucin 16, cell surface associated (**MUC16**), mRNA

```
Query  19     Agggggggggatgg-gggCTGGTTTCTTT  46
              || |||| |||||| ||||||||||||||
Sbjct  23276  AGCGGGGAGGATGGAGGGCTGGTTTCTTT  23248
```

>XM_011528158.2:7-33 PREDICTED: Homo sapiens protein tyrosine phosphatase receptor type S (**PTPRS**), transcript variant X19, mRNA

```
Query  363  TGTGATGACTATCTCTATCCGCGTCGG  389
            ||||||||| ||||| |||| ||||||
Sbjct  33   TGTGATGACCATCTCCATCCTCGTCGG  7
```

>NM_015884.3:3931-3963 Homo sapiens membrane bound transcription factor peptidase, site 2 (**MBTPS2**), mRNA

```
Query  64    TAAAAAGTAATATTGAATAACTAATGATAAATAT  97
             ||||||||| |||||||||  || || |||||||
Sbjct  3931  TAAAAAGTATTATTGAATATTTACTG-TAAATAT  3963
```

>NM_001195216.1:2150-2192 Homo sapiens DENN domain containing 1B (**DENND1B**), transcript variant 4, mRNA

```
Query  78    GAATAACTAATGATAAATATTGCATGGTAGTATAC-GCAAATAAA  121
             ||||  |||||||||||||||  || |||  |||| |||||||||
Sbjct  2150  GAATTTCTAATGATAAATATTTAAT-GTA-AATACTGCAAATAAA  2192
```

```
>NM_152791.4:3622-3640 Homo sapiens zinc finger protein 555 (ZNF555), transcript
variant 1, mRNA
Query  40    TTTCTTTTCTTGCTTGTGT  58
             |||||||||||||||||||
Sbjct  3640  TTTCTTTTCTTGCTTGTGT  3622
```

```
>NM_001184735.1:736-782 Homo sapiens astrotactin 2 (ASTN2), transcript variant 6,
mRNA
Query  108  TATACGCAAATAAATAAATG--ATACATCAATCAATTGTTTAGAGGT  152
            ||| ||||||| ||||||||  || || |||| || ||| || ||||
Sbjct  782  TATCCGCAAATCAATAAATGTAATCCAGCAATAAACTGTGTACAGGT  736
```

M protein, blastn for membrane glycoprotein and hsa transcripts

>QHD43419.1 membrane glycoprotein [Severe acute respiratory syndrome coronavirus 2], **AA-sequence**

MADSNGTITVEELKKLLEQWNLVIGFLFLTWICLLQFAYANRNRFLYIIKLIFLWLLWPVTLACFVLAAVYRINWITGGIA
IAMACLVGLMWLSYFIASFRLFARTRSMWSFNPETNILLNVPLHGTILTRPLLESELVIGAVILRGHLRIAGHHLGRCDIK
DLPKEITVATSRTLSYYKLGASQRVAGDSGFAAYSRYRIGNYKLNTDHSSSSDNIALLVQ

AA-sequence is transcribed to the imaginary nucleotide sequences by 1-letter code, and then, the sequences are used for blastn-search in the human transcriptome (the conditions for the blastn-search are described in the paper):

* Mc transcript, S (Ser) transcription to C (cytidine)

UCACAGCUCUAAUAAUUAAGAUUUGUUUUCGUGUUAUCACAGAGUUAUUAUUUUGUUGCUCUCGUUUCCUAGUAGUCGGUC
UCUCGUUGUUGUCAUUCCUGUUCGCGCUGCUACACAUUUAUCUAGCUUCGCUUACAUUUGCUUUGGAUGUCGAAUGGGAUA
AUCAAUCUCCCGCUCAAAUGCCAGUCGACGUCCACGAGUGAAAUACAACCCCAAUCUUUA

```
>XM_005250915.4:7201-7222 PREDICTED: Homo sapiens RNA binding motif protein 12B
(RBM12B), transcript variant X1, mRNA RBM12B, MGC
Query  39    ACAGAGTTATTATTTTGTTGCT  60
             ||||||||||||||||| ||||
Sbjct  7222  ACAGAGTTATTATTTTGCTGCT  7201
```

```
>XM_005250787.2:2043-2064 PREDICTED: Homo sapiens family with sequence similarity
92 member A (FAM92A), transcript variant X1, mRNA
Query  39    ACAGAGTTATTATTTTGTTGCT  60
             ||||||||||||||||| ||||
Sbjct  2043  ACAGAGTTATTATTTTGCTGCT  2064
```

```
>NM_004716.3:3823-3855 Homo sapiens proprotein convertase subtilisin/kexin type 7
(PCSK7), mRNA PCSK7, LPC, PC7, PC8, SPC7
Query  10    TAATAATTAAGATTTGTTTTCGTGTTATCACAG  42
             ||||  ||||||||||||||  | || ||||||
Sbjct  3823  TAATGCTTAAGATTTGTTTTTCTCTTTTCACAG  3855
```

```
>NM_001001522.2:1926-1958 Homo sapiens transgelin (TAGLN), transcript variant 1,
mRNA TAGLN, SM22, SM22-alpha, SMCC, TAGLN1, WS3-10
Query  10    TAATAATTAAGATTTGTTTTCGTGTTATCACAG  42
             ||||  ||||||||||||||  | || ||||||
```

```
Sbjct  1958  TAATGCTTAAGATTTGTTTTTCTCTTTTCACAG  1926
```

>NM_003048.5:4828-4866 Homo sapiens solute carrier family 9 member A2 (**SLC9A2**), mRNA SLC9A2, NHE2

```
Query  10    TAATAATTAAGATTT--GTTTTCGTGTTATCACAGAGTTAT  48
             |||||||||||||||  |||||| | |||| ||| ||| ||
Sbjct  4866  TAATAATTAAGATTTAAGTTTTCCT-TTAT-ACATAGTCAT  4828
```

>NM_001012754.3:1491-1536 Homo sapiens NHL repeat containing 3 (**NHLRC3**), transcript variant 1, mRNA

```
Query  18    AAGATTTGTTTTCGTGTTATCACAGAGT-TATTATTTTGTTGCTCTC  63
             |||||||||||| || | || | ||| | |  ||||||||||| |||
Sbjct  1491  AAGATTTGTTTTTGTATCATTA-AGAATCTTATATTTTGTTGCCCTC  1536
```

>NM_032900.5:2234-2251 Homo sapiens Rho GTPase activating protein 19 (**ARHGAP19**), transcript variant 1, mRNA

```
Query  10    TAATAATTAAGATTTGTT  27
             ||||||||||||||||||
Sbjct  2251  TAATAATTAAGATTTGTT  2234
```

* Mcr transcript, S (Ser) transcription to C; transcription of reversed protein sequence

```
AUUUCUAACCCCAACAUAAAGUGAGCACCUGCAGCUGACCGUAAACUCGCCCUCUAACUAAUAGGGUAAGCUGUAGGUUUC
GUUUACAUUCGCUUCGAUCUAUUUACACAUCGUCGCGCUUGUCCUUACUGUUGUUGCUCUCUGGCUGAUGAUCCUUUGCUC
UCGUUGUUUUAUUAUUGAGACACUAUUGUGCUUUUGUUUAGAAUUAAUAAUCUCGACACU
```

>NM_001042492.2:5983-6001 Homo sapiens neurofibromin 1 (**NF1**), transcript variant 1, mRNA NF1, NFNS, VRNF, WSS

```
Query  31    GCAGCTGACCGTAAACTCG  49
             |||||||||||||||||||
Sbjct  6001  GCAGCTGACCGTAAACTCG  5983
```

>NM_001102598.2:905-922 Homo sapiens carcinoembryonic antigen related cell adhesion molecule 20 (**CEACAM20**), transcript variant 4S, mRNA
CEACAM20, UNQ9366

```
Query  20   AGTGAGCACCTGCAGCTG  37
            ||||||||||||||||||
Sbjct  905  AGTGAGCACCTGCAGCTG  922
```

>XM_017016985.2:844-861 PREDICTED: Homo sapiens SPARC (osteonectin), cwcv and kazal like domains proteoglycan 2 (**SPOCK2**), transcript variant X2, mRNA
SPOCK2, testican-2

```
Query  126  TTACTGTTGTTGCTCTCT  143
            ||||||||||||||||||
Sbjct  861  TTACTGTTGTTGCTCTCT  844
```

* Mg transcript, S (Ser) transcription to G (guanosine)

```
UCAGAGCUCUAAUAAUUAAGAUUUGUUUUCGUGUUAUCACAGAGUUAUUAUUUUGUUGCUCUCGUUUCCUAGUAGUCGGUC
UCUCGUUGUUGUGAUUCGUGUUCGCGGUGGUACACAUUUAUCUAGCUUCGCUUAGAUUUGCUUUGGAUGUCGAAUGGGAUA
AUCAAUCUCCGGCUGAAAUGCGAGUCGAGGUCCAGGAGUGAAAUACAAGGGGAAUCUUUA
```

--

* Mgr transcript, S (Ser) transcription to G (guanosine); transcription of reversed protein sequence

AUUUCUAAGGGGAACAUAAAGUGAGGACCUGGAGCUGAGCGUAAAGUCGGCCUCUAACUAAUAGGGUAAGCUGUAGGUUUC
GUUUAGAUUCGCUUCGAUCUAUUUACACAUGGUGGCGCUUGUGCUUAGUGUUGUUGCUCUCUGGCUGAUGAUCCUUUGCUC
UCGUUGUUUUAUUAUUGAGACACUAUUGUGCUUUUGUUUAGAAUUAAUAAUCUCGAGACU

>XM_024449397.1:25-61 PREDICTED: Homo sapiens sarcoglycan gamma (**SGCG**), transcript variant X3, mRNA
SGCG, 35DAG, A4, DAGA4, DMDA, DMDA1, LGMD2C, LGMDR5, MAM, SCARMD2, SCG3, gamma-SG

```
Query  152  ATCCTTTGCTCTCGTTGTT-TTATTATTGAGACACTAT  188
            ||||||||||||| || || ||||| || ||| |||||
Sbjct  25   ATCCTTTGCTCTCATTCTTCTTATT-TTCAGAAACTAT  61
```

>NM_001037132.2:1989-2012 Homo sapiens neuronal cell adhesion molecule (**NRCAM**), transcript variant 1, mRNA

```
Query  116   GCGCTTGTGCTTAGTGTTGTTGCTC  140
             ||||| |||||||| ||||||||||
Sbjct  1989  GCGCT-GTGCTTAGCGTTGTTGCTC  2012
```

>NM_001267578.1:3274-3316 Homo sapiens torsin 1A interacting protein 1 (**TOR1AIP1**), transcript variant 1, mRNA
TOR1AIP1, LAP1, LAP1B, LAP1C, LGMD2Y

```
Query  170   TTTATTATTGAGACACTATTGTGCTTTTGTTTAGAATTAATAA  212
             |||||| | || ||||  |   |||||||||| ||||||| ||
Sbjct  3274  TTTATTTTGGACACACCCTGAAGCTTTTGTTTTGAATTAAGAA  3316
```

N protein, blastn for nucleocapsid phosphoprotein and hsa transcripts

>QHD43423.2 nucleocapsid phosphoprotein [Severe acute respiratory syndrome coronavirus 2], **AA-sequence**

MSDNGPQNQRNAPRITFGGPSDSTGSNQNGERSGARSKQRRPQGLPNNTASWFTALTQHGKEDLKFPRGQGVPINTNSSPD
DQIGYYRRATRRIRGGDGKMKDLSPRWYFYYLGTGPEAGLPYGANKDGIIWVATEGALNTPKDHIGTRNPANNAAIVLQLP
QGTTLPKGFYAEGSRGGSQASSRSSSRSRNSSRNSTPGSSRGTSPARMAGNGGDAALALLLLDRLNQLESKMSGKGQQQQG
QTVTKKSAAEASKKPRQKRTATKAYNVTQAFGRRGPEQTQGNFGDQELIRQGTDYKHWPQIAQFAPSASAFFGMSRIGMEV
TPSGTWLTYTGAIKLDDKDPNFKDQVILLNKHIDAYKTFPPTEPKKDKKKKADETQALPQRQKKQQTVTLLPAADLDDFSK
QLQQSMSSADSTQA

AA-sequence is transcribed to the imaginary nucleotide sequences by 1-letter code, and then, the sequences are used for blastn-search in the human transcriptome (the conditions for the blastn-search are described in the paper):

* Nc transcript, S (Ser) transcription to C (cytidine)

UCAAGCAAAGACCGUCUGGCCACCGCAAAGAGCGCGCAAGGCAGUCAACCCGUCCUCAAGAAAUAUCGGAGUCUACACCCA
AAUGAAGGCCGGUGGGAGAUAAUCCGGAUAAUGCGCACGUCAGCAAAGUUGUCCAGCUACCAAAUGCGACCAACCUUUAUC
AGCCUCAGUACAGCGGGCACCCGCCCGCGACCGACCCGCCGGCCCCGUCGAGGACCUCUUUUAGUAAUACAUCGAGAAAAG
ACUCAACCCACCAACGAAGCCCACAAUCACUGGGGCAACAGAUGAAAUUGAGCAAAAGCAUCAUCCCCCCUUGUCGUGUAU
CCCGCGUCACGCUAUAAAACAUAAAUUUUAAAUACAACUCCCACAAAAAAACAACACUCAGAAAAACUCUUCCCAUAAUCA
AUAACUCCCACCAC

>NM_001082538.2:2002-2034 Homo sapiens tectonic family member 1 (**TCTN1**), transcript variant 1, Mrna

```
Query  346   TAAATTTTAAATACAACTCCCACaaaaaaaCAA  378
             ||||||||| ||||||||  || ||||||| ||
Sbjct  2002  TAAATTTTATATACAACTAGCAAAAAAAAAAAA  2034
```

>NM_203391.3:2694-2713 Homo sapiens glycerol kinase (**GK**), transcript variant 1, mRNA

```
Query  338   ATAAAACATAAATTTTAAAT  357
             ||||||||||||||||||||
Sbjct  2713  ATAAAACATAAATTTTAAAT  2694
```

>NM_003643.3:2323-2367 Homo sapiens glial cells missing homolog 1 (**GCM1**), mRNA

```
Query  336   CTATAAAACATAAATTTTAAATACAACTCCCACaaaaaaaCAACA  380
             ||| |||||||| |||||| | || | || |||||  | ||||||
Sbjct  2367  CTAGAAAACATATATTTTACACACTAGTCTCACAACTAGACAACA  2323
```

* Ncr transcript, S (Ser) transcription to C; transcription of reversed protein sequence

CACCACCCUCAAUAACUAAUACCCUUCUCAAAAAGACUCACAACAAAAAAACACCCUCAACAUAAAUUUUAAAUACAAAAU
AUCGCACUGCGCCCUAUGUGCUGUUCCCCCCUACUACGAAAACGAGUUAAAGUAGACAACGGGGUCACUAACACCCGAAGC
AACCACCCAACUCAGAAAAGAGCUACAUAAUGAUUUUCUCCAGGAGCUGCCCCGGCCGCCCAGCCAGCGCCCGCCCACGGG
CGACAUGACUCCGACUAUUUCCAACCAGCGUAAACCAUCGACCUGUUGAAACGACUGCACGCGUAAUAGGCCUAAUAGAGG
GUGGCCGGAAGUAAACCCACAUCUGAGGCUAUAAAGAACUCCUGCCCAACUGACGGAACGCGCGAGAAACGCCACCGGUCU
GCCAGAAACGAACU

>XM_011522615.2:1400-1433 PREDICTED: Homo sapiens lipase maturation factor 1 (**LMF1**), transcript variant X7, mRNA Record removed.

```
Query  213   CCCGGCCGCCCAGCCAGCGCCCGCCCACGGGCGACAT  249
             |||||||||||||||||| |   |||||||| | |||
Sbjct  1400  CCCGGCCGCCCAGCCAGCAC---CCCACGGGAGCCAT  1433
```

>NM_001304808.2 Homo sapiens bromodomain containing 1 (**BRD1**), transcript variant 1, mRNA

```
Query  207   AGCTGCCCCGGCCGCCCAGCCAGCGCCCGCCCACGGG  243
             |||||  || || || |||||||| || |||||||||
Sbjct  2706  AGCTGAGCCAGCAGCACAGCCAGCCCCTGCCCACGGG  2742
```

>NM_001282062.1:155-181 Homo sapiens double C2 domain alpha (**DOC2A**), transcript variant 1, mRNA

```
Query  211  GCCCCGGCCGCCCAGCCAGCGCCCGCC  237
            ||| | ||||||||||| |||||||||
Sbjct  155  GCCGCCGCCGCCCAGCCTGCGCCCGCC  181
```

>NM_005094.3:114-141 Homo sapiens solute carrier family 27 member 4 (**SLC27A4**), mRNA

```
Query  210  TGCCCCGGCCGCCCAGCCAGCGCCCGCCC  238
            || ||||||||||| |||||| |||||||
Sbjct  141  TGGCCCGGCCGCCCCGCCAGC-CCCGCCC  114
```

>NM_178161.2:221-247 Homo sapiens pancreas associated transcription factor 1a (**PTF1A**), mRNA

```
Query  212  CCCCGGCCGCCCAGCCAGCGCCCGCCC  238
```

```
             |||||||||||| || ||||| |||||
Sbjct  221  CCCCGGCCGCCCCGCTAGCGCTCGCCC  247
```

>NM_001453.2:571-612 Homo sapiens forkhead box C1 (**FOXC1**), mRNA

```
Query  200  CTCCAGGAGCTGCCCCGGCCGCCCAGCCAGCGCCCGCCCACG  241
            ||| |||||| ||||| |||   | |||||| ||||||| ||
Sbjct  571  CTCAAGGAGCCGCCCCCGCCCGGCCGCCAGCCCCCGCCCGCG  612
```

>NM_003685.2:2099-2127 Homo sapiens KH-type splicing regulatory protein (**KHSRP**), transcript variant 1, mRNA

```
Query  201   TCCAGGAGCTGCCCCGGCCGCCCAGCCAG  229
             |||||||||| |||| | | |||||||||
Sbjct  2099  TCCAGGAGCTCCCCCAGGCTCCCAGCCAG  2127
```

>NM_032496.3:2012-2035 Homo sapiens Rho GTPase activating protein 9 (**ARHGAP9**), transcript variant 1, mRNA

```
Query  196   TTTTCTCCAGGAGCTGCCCCGGCC  219
             |||||||| ||||||||||| |||
Sbjct  2012  TTTTCTCCGGGAGCTGCCCCAGCC  2035
```

>NM_017798.3:150-183 Homo sapiens YTH N6-methyladenosine RNA binding protein 1 (**YTHDF1**), mRNA

```
Query  202  CCAGGAGCTGCCCCGGCCGCCCAGCCAGCGCCCG  235
            |||| ||| ||| | ||||||| ||| |||||||
Sbjct  150  CCAGCAGCCGCCGCCGCCGCCCGGCCGGCGCCCG  183
```

* Ng transcript, S (Ser) transcription to G (guanosine)

```
UGAAGCAAAGACCGUCUGGCGAGCGGAAAGAGGGCGGAAGGCAGUCAACCGGUCCUCAAGAAAUAUCGGAGUCUACAGGCA
AAUGAAGGCCGGUGGGAGAUAAUGCGGAUAAUGCGCACGUCAGCAAAGUUGUCCAGCUACCAAAUGCGACCAACCUUUAUC
AGCCUCAGUACAGGGGGGACGGGGGGGGGAGGGAGCCGGGGGCGCCGUCGAGGACCUCUUUUAGUAAUAGAUGGAGAAAAG
ACUCAAGCCACGAACGAAGCCCACAAUCACUGGGGCAACAGAUGAAAUUGAGCAAAAGCAUCAUCCGCGCUUGUGGUGUAU
CCGGCGUCACGCUAUAAAACAUAAAUUUUAAAUACAACUCCCACAAAAAAACAACACUCAGAAAAACUCUUCCCAUAAUGA
AUAAGUGGCAGCAC
```

>XM_011522356.1:597-629 PREDICTED: Homo sapiens coiled-coil domain containing 78 (**CCDC78**), transcript variant X1, mRNA

```
Query  162  CAGCCTCAGTACAgggggacgggggggggagg  194
            |||||||||| || ||||||||||||| | |||
Sbjct  597  CAGCCTCAGTGCATGGGGGACGGGGGGTGCAGG  629
```

>NM_003773.4:1230-1257 Homo sapiens hyaluronidase 2 (**HYAL2**), transcript variant 1, mRNA

```
Query  307   TCCGCGCTTGTGGTGTATCCGGCGTCAC  334
             |||| |||||||||||| || |||||||
Sbjct  1257  TCCGTGCTTGTGGTGTACCCCGCGTCAC  1230
```

>NM_017805.2:203-229 Homo sapiens Ras interacting protein 1 (**RASIP1**), mRNA

```
Query  14   GTCTGGCGAGCGGAAAGAGGGCGGAAG  40
            |||||| || ||||| |||||||||||
Sbjct  203  GTCTGGTGAACGGAAGGAGGGCGGAAG  229
```

>NM_015177.1:130-169 Homo sapiens deltex E3 ubiquitin ligase 4 (**DTX4**), transcript

```
Query  178  gggacgggggggggagggagccgggggCGCCGTCGAGGACC  218
            ||| ||| |||| | ||| || |||||||| ||||||| ||
```

```
Sbjct  130  GGGGCGGCGGGGCGCGGG-GCAGGGGGCGCGGTCGAGGCCC  169
```

>NM_006412.3:51-86 Homo sapiens 1-acylglycerol-3-phosphate O-acyltransferase 2 (**AGPAT2**), transcript variant 1, mRNA

```
Query  178  ggga-cgggggggggagggagccgggggCGCCGTCG  212
            |||| |||| | ||||| |||| || ||||||||||
Sbjct  51   GGGAGCGGGAGCGGGAGCGAGCTGGCGGCGCCGTCG  86
```

* Ngr transcript, S (Ser) transcription to G (guanosine); transcription of reversed protein sequence

CACGACGGUGAAUAAGUAAUACCCUUCUCAAAAAGACUCACAACAAAAAAACACCCUCAACAUAAAUUUUAAAUACAAAAU
AUCGCACUGCGGCCUAUGUGGUGUUCGCGCCUACUACGAAAACGAGUUAAAGUAGACAACGGGGUCACUAACACCCGAAGC
AAGCACCGAACUCAGAAAAGAGGUAGAUAAUGAUUUUCUCCAGGAGCUGCCGCGGGGGCCGAGGGAGGGGGGGGGCAGGGG
GGACAUGACUCCGACUAUUUCCAACCAGCGUAAACCAUCGACCUGUUGAAACGACUGCACGCGUAAUAGGCGUAAUAGAGG
GUGGCCGGAAGUAAACGGACAUCUGAGGCUAUAAAGAACUCCUGGCCAACUGACGGAAGGCGGGAGAAAGGCGAGCGGUCU
GCCAGAAACGAAGU

>NM_016639.2:184-214 Homo sapiens TNF receptor superfamily member 12A (**TNFRSF12A**), mRNA

```
Query  200  CTCCAGGAGCTGCCGCgggggccgagggagg  230
            ||||||||||||||||||| || | ||| ||
Sbjct  214  CTCCAGGAGCTGCCGCGGGAGCAGGGGGCGG  184
```

>XM_017006347.1:1610-1649 PREDICTED: Homo sapiens interleukin 1 receptor accessory protein (**IL1RAP**), transcript variant X2, mRNA

```
Query  23    CCTTCTCAAAAAGACTCACAACaaaaaaaCACCCTCAACA  62
             |||||||||||| |   |||||||||||| |  | |||||
Sbjct  1649  CCTTCTCAAAAAAAACAACAACAAAAAAAAAAACACAACA  1610
```

>NM_021982.2:5605-5637 Homo sapiens SEC24 homolog A, COPII coat complex component (**SEC24A**), transcript variant 1, mRNA

```
Query  30    AAAAAGACTCACAACaaaaaaaCACCCTCAACAT  63
             ||||||| ||| ||| |||||||||  |||||||
Sbjct  5637  AAAAAGAATCA-AACTAAAAAAACACATTCAACAT  5605
```

>NM_001858.5:6389-6414 Homo sapiens collagen type XIX alpha 1 chain (**COL19A1**), mRNA

```
Query  58    CAACATAAAT-TTTAAATACAAAATA  82
             ||||||| || |||||||||||||||
Sbjct  6389  CAACATACATGTTTAAATACAAAATA  6414
```

>NM_022098.3:5289-5344 Homo sapiens X-prolyl aminopeptidase 3 (**XPNPEP3**), transcript variant 1, mRNA

```
Query  23    CCTTCTCAAAAAGACTCACAACaaaaaaaCACCCTCAACATAAATTTTAAATACAAAA  80
             || ||||||||| |   | ||||||||||| ||| ||| | ||  |||||| | ||||
Sbjct  5344  CCATCTCAAAAAAAAAAAAACAAAAAAAC-CCCACAA-AAAACCTTTAAAAAAAAAA5289
```
